# Genetics of early-life head circumference and genetic correlations with neurological, psychiatric and cognitive outcomes

**DOI:** 10.1186/s12920-022-01281-1

**Published:** 2022-06-04

**Authors:** Suzanne Vogelezang, Jonathan P. Bradfield, the Early Growth Genetics Consortium, Suzanne Vogelezang, Jonathan P. Bradfield, Stefan Johansson, Evie Stergiakouli, Elisabeth Thiering, Craig E. Pennell, Tarunveer S. Ahluwalia, Ville Karhunen, Markus Scholz, Xueping Liu, Carmen Iñiguez, Olli T. Raitakari, Jonas Bacelis, Theresia M. Schnurr, Timo A. Lakka, Ioanna Ntalla, Mads V. Lind, Lotte Lauritzen, Sauli Herrala, Frederick T. J. Lin, Christine Frithioff-Bøjsøe, Robin N. Beaumont, Mohammed H. Zafarmand, Peter Rzehak, Jose R. Bilbao, Nella Junna, Judith M. Vonk, Sheryl L. Rifas-Shiman, Vimala D. Janjanam, Maria J. Knol, Shweta Ramdas, Lawrie Beilin, Klaus Bønnelykke, Maribel Casas, Johan G. Eriksson, Joaquin Escribano, Tavia E. Evans, Ulrike Gehring, Frank Geller, Veit Grote, Darek Gruszfeld, Hakon Hakonarson, Andrew T. Hattersley, Christian T. Have, M. Geoffrey Hayes, Joachim Heinrich, Øvind Helgeland, Jhon Holloway, Raimo Joro, Julius Juodakis, Bridget A. Knight, Bert Koletzko, Antje Körner, Jean-Paul Langhendries, Jaakko T. Leinonen, Virpi Lindi, Morten A. V. Lund, Stephen Lye, Mads Melbye, Kim F. Michaelsen, Camilla S. Morgen, Harri Niinikoski, Katja Pahkala, Kalliope Panoutsopoulou, Oluf Pedersen, Fernando Rivadeneira, Loreto Santa-Marina, Barbera D. C. Schaik, Denise Scholtens, Sylvain Sebert, Ibon Tamayo, Nicholas J. Timpson, Maties Torrent, André G. Uitterlinden, Marc Vaudel, Elvira Verduci, Rebecca Vinding, Mandy Vogel, Eleftheria Zeggini, Christopher Brown, Hieab H. H. Adams, Wilfried Karmaus, Marie-France Hivert, Gerard H. Koppelman, Elisabeth Widén, Nora Fernandez-Jimenez, Melanie Waldenberger, Tanja G. M. Vrijkotte, Rachel M. Freathy, Jens-Christian Holm, William L. Lowe, Niels Grarup, Torben Hansen, George V. Dedoussis, Mustafa Atalay, Ellen A. Nohr, Bo Jacobsson, Niina Pitkänen, Martine Vrijheid, Bjarke Feenstra, Wieland Kiess, Marjo-Riita Jarvelin, Hans Bisgaard, Carol Wang C, Marie Standl, Mark I. McCarthy, Beate St Pourcain, Pål R. Njølstad, Struan F. A. Grant, Janine F. Felix, Vincent W. V. Jaddoe

**Affiliations:** 1 The Generation R Study Group, Erasmus MC, University Medical Center Rotterdam, Rotterdam, The Netherlands; 2 Department of Pediatrics, Erasmus MC, University Medical Center Rotterdam, Rotterdam, The Netherlands; 3 Department of Epidemiology, Erasmus MC, University Medical Center Rotterdam, Rotterdam, The Netherlands; 4 Division of Human Genetics, Center for Applied Genomics, Children’s Hospital of Philadelphia, Philadelphia, PA USA; 5 Quantinuum Research LLC, San Diego, CA USA; 6 Department of Pediatrics, Perelman School of Medicine, University of Pennsylvania, Philadelphia, PA USA; 7 Division of Endocrinology and Diabetes, The Children’s Hospital of Philadelphia, Philadelphia, PA USA; 8 Division of Human Genetics, Center for Spatial and Functional Genomics, Children’s Hospital of Philadelphia, Philadelphia, PA USA

**Keywords:** Head circumference, Genome-wide association study, Genetic correlations, Infancy

## Abstract

**Background:**

Head circumference is associated with intelligence and tracks from childhood into adulthood.

**Methods:**

We performed a genome-wide association study meta-analysis and follow-up of head circumference in a total of 29,192 participants between 6 and 30 months of age.

**Results:**

Seven loci reached genome-wide significance in the combined discovery and replication analysis of which three loci near *ARFGEF2*, *MYCL1*, and *TOP1*, were novel. We observed positive genetic correlations for early-life head circumference with adult intracranial volume, years of schooling, childhood and adult intelligence, but not with adult psychiatric, neurological, or personality-related phenotypes.

**Conclusions:**

The results of this study indicate that the biological processes underlying early-life head circumference overlap largely with those of adult head circumference. The associations of early-life head circumference with cognitive outcomes across the life course are partly explained by genetics.

**Supplementary Information:**

The online version contains supplementary material available at 10.1186/s12920-022-01281-1.

## Background

Head circumference is a complex trait, commonly used as an indicator of brain volume during development and associated with child and adult intelligence [1–3]. It is also used a s measure of skeletal growth in fetal life, at birth and in early childhood [4, 5]. Twin studies show heritability estimates ranging from 75 to 90%, which are consistent across the life course [6]. Large genome-wide association studies (GWAS) have identified multiple loci associated with child and adult head circumference, intracranial volume and brain volume [7–10]. Heritability estimates from GWAS range from 10 to 31% [8]. However, only two genetic loci associated with head circumference between 6 and 30 months have been identified so far [11]. Identifying additional genetic loci related to early-life head circumference may contribute towards our understanding of early brain development. This is important since observational studies have associated early brain development with several neurological and psychiatric diseases, such as Alzheimer’s disease, schizophrenia and autism [12–17]. The underlying mechanisms are poorly understood. Both genetics and environmental factors play a role [18]. Additionally, the shared genetic contribution between early-life head circumference and later-life outcomes is yet unknown. Unravelling this shared genetic contribution may help us to better understand the etiology of later-life outcomes related to early-life head circumference.

We examined the genetic background of early-life head circumference by performing a two-stage GWAS meta-analysis comprising 25 studies with a combined sample size of 29,192 European-ancestry participants between 6 and 30 months of age. We also examined genetic correlations of early-life head circumference with anthropometrics, brain volume-related, neurological, psychiatric, cognitive, and personality related traits.

## Methods

### Study design

We conducted a two-stage meta-analysis in children of European ancestry to identify genetic loci associated with birth and early-life head circumference. Sex- and age-adjusted standard deviation scores (SDS) were created for head circumference between 6 to 30 months (closest to 18 months, if multiple measurements were available) using Growth Analyzer 3.0 across all studies [19]. In the case of twin pairs and siblings, only one of each twin or sibling pair was included, either randomly or based on genotyping or imputation quality.

In the discovery stage, we performed a meta-analysis of early-life head circumference in 21 studies (N = 22,279), including the Amsterdam Born Children and their Development-Genetic Enrichment Study (ABCD, N = 1018), the Avon Longitudinal Study of Parents and Children (ALSPAC, N = 3960), Children’s Hospital of Philadelphia (CHOP, N = 856), the Copenhagen Prospective Studies on Asthma in Childhood 2000 (COPSAC2000, N = 325) and 2010 (COPSAC2010, N = 603), the Danish National Birth Cohort- preterm birth study (DNBC-PTB, N = 508), the Generation R Study (GenerationR, N = 2299), the Danish National Birth Cohort—the Genetics of Overweight Young Adults offspring study (DNBC GOYA-offspring, N = 230), the INfancia y Medio Ambiente [Environment and Childhood] Project, with two subcohorts that were entered into the meta-analysis separately (INMA-Sabadell and Valencia subcohort and INMA Menorca subcohort, N = 550), the German Infant Study on the influence of Nutrition Intervention PLUS environmental and genetic influences on allergy development & Influence of life-style factors on the development of the immune system and allergies in East and West Germany (GINIplus&LISA, N = 1455), the Leipzig Research Center for Civilization Diseases—Child study (LIFE-Child, N = 1365), the Norwegian Mother Child Cohort (MoBa, N = 836), the Northern Finland Birth Cohort 1966 (NFBC 1966, N = 4603), the Northern Finland Birth Cohort 1986 (NFBC 1986, N = 826), the Physical Activity and Nutrition in Children Study (PANIC, N = 372), the Raine Study (Raine Study, N = 1373), the Småbørns Kost Og Trivsel study, including two subcohorts (SKOT 1, N = 170 and SKOT 2, N = 98), the Special Turku Coronary Risk factor Intervention Project (STRIP, N = 505), and the TEENs of Attica: Genes and Environment (TEENAGE, N = 327).

In the replication stage of early-life head cirvumference analysis, we included 4 studies (N = 6913): 319 additional children from the INfancia y Medio Ambiente [Environment and Childhood] (INMA) Project (INMA-Gipuzkoa subcohort), 5644 additional children from the Norwegian Mother and Child Cohort (MoBa), the European Childhood Obesity Project (CHOP Study, N = 366), and the Exeter Family Study of Childhood Health (EFSOCH, N = 584). Characteristics of discovery and replication studies can be found in Additional file 2: Table S1. The study design of birth head circumference can be found in the Additional file 1.

### Study-level analyses

Genome-wide association analyses were first run in all discovery cohorts for birth and early-life head circumference separately. Studies used high-density Illumina or Affymetrix Single Nucleotide Polymorphism (SNP) arrays, followed by imputation to the 1000 Genomes Project or Haplotype Reference Consortium (HRC). Before imputation, studies applied study specific quality filters on sample and SNP call rate, minor allele frequency and Hardy–Weinberg disequilibrium (see Additional file 2: Table S1 for details). Linear regression models assuming an additive genetic model were run in each study, to assess the association of each SNP with SDS head circumference, adjusting for principal components if this was deemed needed in the individual studies. As SDS head circumference is age and sex specific, no further adjustments were made. Before the meta-analysis, we applied quality filters to each study, filtering out SNPs with a minor allele frequency (MAF) below 1% and SNPs with poor imputation quality (MACH r2_hat ≤ 0.3, IMPUTE proper_info ≤ 0.4 or info ≤ 0.4).

### Meta-analysis

We performed fixed-effects inverse-variance weighted meta-analysis of all discovery samples using Metal [20]. Genomic control was applied to every study before the meta-analysis. Individual study lambdas before genomic control ranged from 0.99 to 1.03 (Additional file 2: Table S1). The lambda of the discovery meta-analysis was 1.02. Linkage Disequilibrium (LD) score regression analysis showed an intercept of 1.0, indicating that the slight inflation was mainly caused by polygenicity of early-life head circumference and not by population stratification, cryptic relatedness or other confounders [21, 22]. After the meta-analysis, we excluded SNPs for which information was available in less than 50% of the studies or in less than 50% of the total sample size.

Genome-wide Complex Trait Analysis (GCTA) was used to select the independent SNPs for each locus [23]. We performed conditional analyses based on summary-level statistics and LD estimation between SNPs from the Generation R Study as a reference sample to select independently associated SNPs on the basis of conditional *P* values [23]. For early-life head circumference, 27 genome-wide significant or suggestive loci (*P* values < 5 × 10^–8^ and *P* values < 5 × 10^−6^, respectively) were taken forward for replication in the 4 replication cohorts. Fixed-effects inverse variance weighted meta-analysis was performed for these 27 SNPs combining the discovery samples and all replication samples, giving a combined analysis beta, standard error and *P* value (Table 1). SNPs that reached genome-wide significance (*P* value < 5 × 10^–8^) in the combined analysis were considered to be significantly associated with SDS-head circumference. For birth head circumference, SNPs were taken forward for replication, using the same methodology.

**Table 1:** Results of the 27 SNPs with *P* values < 5 × 10^–6^ in the discovery phase

SNP	CHR	Position	Nearest gene	EA/non_EA	*P* value discovery	*P* value replication	EAF_a_	Beta (SE) combined	*P* value combined	Heterogeneity I^2^ combined analysis
rs8756_b, c, d_	12	66,359,752	*HMGA2*	C/A	5.18 × 10^–12^	4.62 × 10^–3^	0.50	0.065 (0.009)	**1.84 × 10**^**–13**^	0
rs9795522_b_	12	123,730,935	*C12orf65*	C/A	3.20 × 10^–9^	3.86 × 10^–5^	0.23	0.072 (0.010)	**7.37 × 10**^**–12**^	0
rs10883848_d_	10	104,973,061	*NT5C2*	A/G	3.06 × 10^–6^	0.03	0.35	0.058 (0.010)	**9.99 × 10**^**–10**^	54.8
rs6095360	20	47,532,536	*ARFGEF2*	G/A	3.53 × 10^–8^	6.19 × 10^–4^	0.32	0.055 (0.009)	**4.39 × 10**^**–9**^	36.6
rs3134614	1	40,363,054	*MYCL1*	C/G	4.31 × 10^–6^	7.75 × 10^–4^	0.88	0.075 (0.013)	**1.43 × 10**^**–8**^	0
rs6016511	20	39,709,255	*TOP1*	T/C	4.55 × 10^–7^	0.02	0.68	0.052 (0.010)	**4.02 × 10**^**–8**^	0
rs7792211_d_	7	50,707,141	*GRB10*	G/A	7.62 × 10^–7^	0.01	0.42	0.051 (0.009)	**4.43 × 10**^**–8**^	11.3
rs1490384_c, d_	6	126,851,160	*CENPW*	T/C	5.46 × 10^–8^	0.14	0.51	0.049 (0.009)	5.70 × 10^–8^*	43.9
rs116536930	17	43,855,156	*CRHR1*	G/T	4.19 × 10^–8^	0.53	0.51	0.055 (0.011)	2.27 × 10^–7^*	40.1
rs72952297	2	180,379,814	*ZNF385B*	T/G	2.07 × 10^–6^	0.11	0.93	0.087 (0.018)	9.19 × 10^–7^*	0
rs12350281	9	20,637,773	*MLLT3*	G/A	4.22 × 10^–6^	0.03	0.05	0.152 (0.031)	9.98 × 10^–7^*	43.1
rs7658192	4	140,023,559	*ELF2*	T/G	2.29 × 10^–7^	0.46	0.33	0.046 (0.010)	1.70 × 10^–6^*	62.9
rs143094271_e_	17	7,463,102	*TNFSF12-TNFSF13*	A/G	5.96 × 10^–7^	0.44	0.02	0.133 (0.028)	2.02 × 10^–6^*	33.7
rs1014098	1	119,458,215	*TBX15*	C/T	2.67 × 10^–6^	0.17	0.79	0.051 (0.011)	2.05 × 10^–6^*	30.3
rs77690628	8	26,189,223	*PPP2R2A*	A/T	1.02 × 10^–6^	0.31	0.07	0.080 (0.017)	3.26 × 10^–6^*	24.6
rs9853018	3	141,101,961	*ZBTB38*	T/C	5.41 × 10^–7^	0.62	0.45	0.040 (0.009)	5.30 × 10^–6^	38.4
rs4674101	2	217,487,622	*RPL37A*	A/G	8.66 × 10^–7^	0.57	0.31	0.043 (0.010)	6.56 × 10^–6^	65.3
rs150266910	1	23,442,265	*LUZP1*	C/T	1.42 × 10^–6^	0.65	0.83	0.052 (0.012)	1.04 × 10^–5^	58.2
rs13067734	3	141,963,015	*GK5*	A/C	2.83 × 10^–6^	0.55	0.52	0.037 (0.009)	1.22 × 10^–5^	26.5
rs17223831	14	90,601,920	*KCNK13*	A/G	1.24 × 10^–6^	0.18	0.14	0.067 (0.015)	1.30 × 10^–5^	67.6
rs62580922	9	125,631,594	*RC3H2*	C/T	1.13 × 10^–8^	0.35	0.07	0.072 (0.017)	1.52 × 10^–5^	77.8
rs2448415	1	93,469,310	*FAM69A*	A/G	1.97 × 10^–6^	0.81	0.47	0.037 (0.009)	2.56 × 10^–5^	60.7
rs261752	5	39,328,506	*C9*	A/G	1.39 × 10^–6^	0.70	0.55	0.035 (0.009)	7.63 × 10^–5^	63.7
rs72979897	6	140,881,181	*MIR3668*	T/A	4.13 × 10^–6^	0.88	0.04	0.100 (0.025)	8.26 × 10^–5^	50.6
rs503783	6	161,959,649	*PARK2*	T/C	2.98 × 10^–6^	0.66	0.27	0.037 (0.010)	1.59 × 10^–4^	74.3
rs113397574	5	31,968,320	*PDZD2*	T/C	4.56 × 10^–6^	0.61	0.02	0.118 (0.032)	2.40 × 10^–4^	58.5
rs77535478	9	77,533,287	*TRPM6*	A/G	1.28 × 10^–6^	0.39	0.04	0.081 (0.026)	2.05 × 10^–3^	75.4

Bolded *P* values indicate genome-wide significance in the combined analysis

*CHR* chromosome, *EA* effect allele, *EAF* effect allele frequency, *SE* standard error

*Genome-wide suggestive loci

^a^From combined analysis

^b^Locus previously reported for infant head circumference [11]

^c^Locus previously reported for adult intracranial volume [7, 9]

^d^Locus previously reported for adult head brain volume [8]

^e^Locus previously reported for childhood and adult head circumference and intracranial volume [10]

### Functional mapping and annotation of genetic associations (FUMA)

To obtain predicted functional consequences for the SNPs that reached genome-wide significance in the combined meta-analysis, we used SNP2FUNC in FUMA, a web-based platform to facilitate and visualize functional annotation of GWAS results [24]. To annotate the nearest genes of the seven SNPs in biological context, we used the GENE2FUNC option in FUMA, which provides hypergeometric tests of enrichment of the list of nearest genes in 53 GTEx tissue-specific gene expression sets [24, 25]. We used GENE2FUNC for two sets of genes: 1. Nearest genes of seven SNPs; 2. Genes located in a region of 500 kb to either side of the 7 SNPs [24].

### Colocalization analysis

We used Bayesian colocalization analysis to examine evidence for colocalization between early-life head circumference and eQTL signals (GTEx v7). Colocalization analyses were conducted using the R package coloc, https://cran.r-project.org/web/packages/coloc, as described previously [26]. Briefly, in each of the GTEx v7 tissues, all cis-eQTLs at FDR < 5% were identified. For each eQTL, GWAS summary statistics were extracted for all SNPs that were present in > 50% of the studies and > 50% of the total sample size and that were in common to both GWAS and eQTL studies, within 1 MB of the transcription start site of the gene. For each such locus, colocalization analyses were done with default parameters, testing the following hypotheses [26]:

- H_0_: No association with either trait;
- H_1_: Association with early-life head circumference only;
- H_2_: Association with gene expression only;
- H_3_: Association with early-life head circumference and gene expression, two distinct causal variants;
- H_4_: Association with early-life head circumference and gene expression, one shared causal variant.

Support for each hypothesis was quantified in terms of posterior probabilities, defined at SNP level and indicated by PP_0_, PP_1_, PP_2_, PP_3_ or PP_4_, corresponding to the five hypotheses and measuring how likely these hypotheses are. In most pairs, no evidence for association was found with either trait. In case association was observed, it was mostly with a single trait. To define colocalization we used restriction to pairs of early-life head circumference and eQTL signals with a high posterior probability for colocalization, indicated by a PP4/(PP3 + PP4) > 0.9.

### The Database for Annotation, Visualization and Integrated Discovery (DAVID)

To explore biological processes, we used DAVID, with the seven nearest genes as input, using the Kyoto Encyclopedia of Genes and Genomes (KEGG) database [27, 28].

#### Linkage-disequilibrium score regression

The use of LD score regression to estimate genetic correlations between two phenotypes has been described in detail previously [29]. Briefly, LD score is a measure of how much a genetic variation is tagged by each variant. A high LD score indicates that a variant is in high LD with many nearby polymorphisms. Variants with high LD scores are more likely to contain true signals and have a higher chance of overlap with genuine signals between GWAS. To estimate LD scores, summary statistics from GWAS meta-analysis are used to calculate the cross-product of test statistics of per SNP, which is regressed on the LD score. The slope of the regression is a function of the genetic covariance between traits [29]:

$$E(z_{1j} z_{2j} ) = \frac{{\sqrt {N_{1} N_{2} } \rho_{g} }}{M}l_{j} + \frac{{\rho N_{s} }}{{\sqrt {N_{1} N_{2} } }}$$

where *N*_*i*_ is the sample size of study *i*, *ρ*_*g*_ is the genetic covariance, *M* is the number of SNPs in the reference panel with a MAF between 5 and 50%, *l*_*j*_ is the LD score for SNP *j*, *N*_*s*_ quantifies the number of individuals that overlap both studies, and ρ is the phenotypic correlation amongst the *N*_*s*_ of overlapping samples. A sample overlap or cryptic relatedness between samples only affects the intercept from the regression but not the slope. Thus, estimates are robust even in presence of sample overlap when comparing traits across distinct GWAS populations. Estimates of genetic covariance are therefore not biased by overlapping samples. Similarly, in case of population stratification, the intercept is affected but it has only minimal impact on the slope since population stratification does not correlate with LD between variants.

Because of the correlation between the imputation quality and LD score, imputation quality is a confounder for LD score regression. Therefore, SNPs were excluded according to the following criteria: MAF < 0.01 or INFO ≤ 0.9. The filtered GWAS results were uploaded on the online webtool, a web service with many GWAS meta-analyses available on which LD score regression has been implemented by the developers of the LD score regression method. In case multiple GWAS meta-analyses were available for the same phenotype, the genetic correlation with early-life head circumference was estimated using the most recent meta-analysis. Genetic correlations are shown in Fig. 3 and Additional file 1: Table S7.

#### Genetic risk score and percentage of variance explained

We combined the seven genome-wide significant SNPs from the combined meta-analysis into a Genetic Risk Score (GRS) by summing up the number of alleles that increase the SDS head circumference, weighted by the effect sizes from the combined meta-analysis. The GRS was rescaled to a range from 0 to 14, which is the maximum number of head circumference SDS increasing alleles and rounded to the nearest integer. Linear regression analysis was used to examine the associations of the risk score with head circumference and intracranial volume at different ages. For these analyses data from the Generation R Study and UK Biobank were used. When calculating the risk score for the Generation R study, effect estimates from the combined meta-analysis were used after excluding Generation R from the meta-analysis. The variance explained was estimated by the adjusted R^2^ of the models.

## Results

### Identification of genetic loci associated with early-life head circumference

**Fig. 1:**
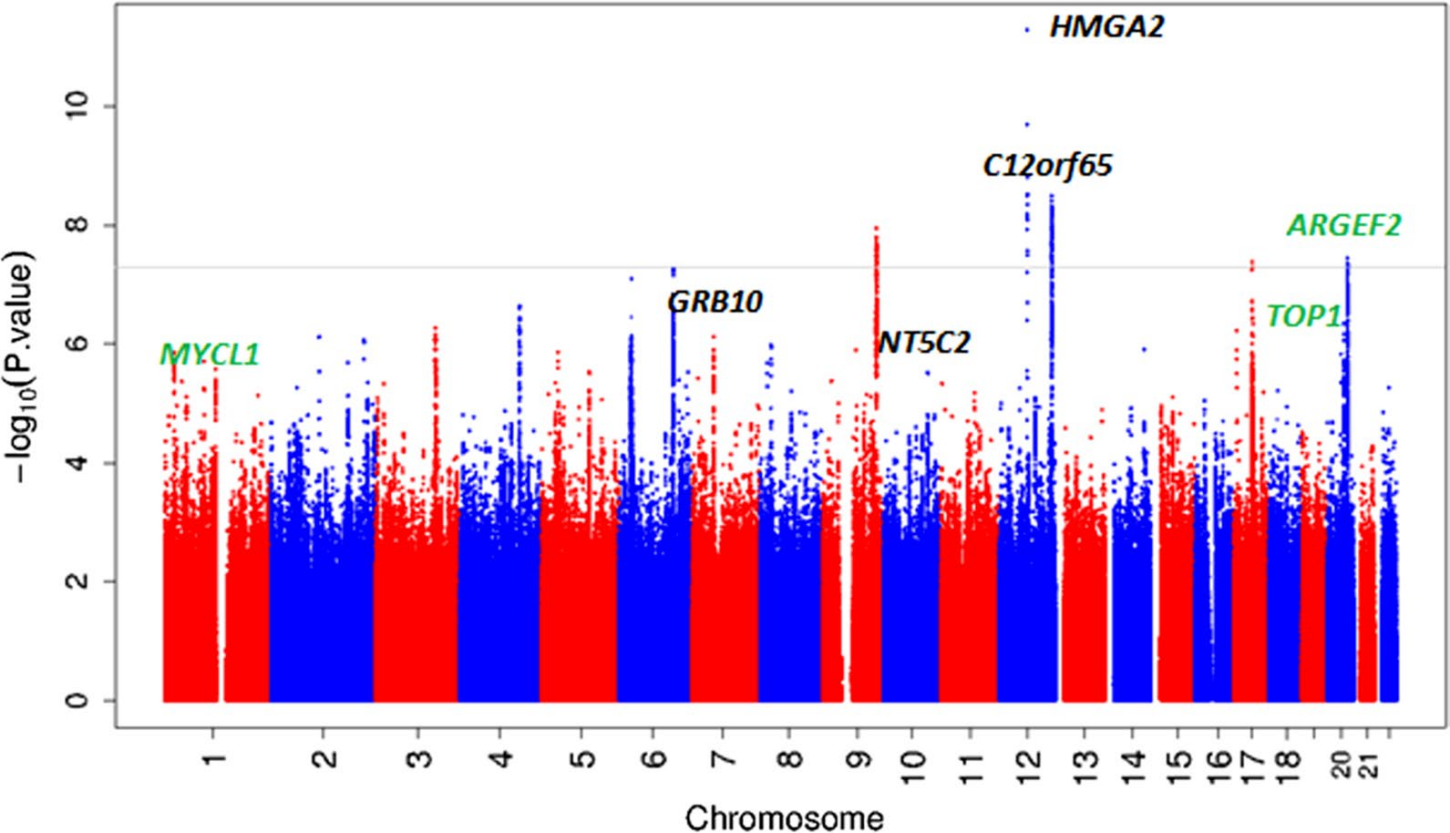
Manhattan plot of results of the discovery meta-analysis of 21 GWAS. On the x-axis the chromosomes are shown. On the y-axis the − log 10 of the *P* value is shown. Novel SNPs are shown in green. Known SNPs are shown in black. The genome wide significance cutoff of 5 × 10^–8^ is represented by the grey dotted line

Individual study characteristics are shown in Additional file 2: Table S1. In the discovery stage, we performed a fixed-effects inverse variance-weighted meta-analysis including data imputed to the 1000 Genomes or the Haplotype Reference Consortium (HRC) reference panels from 21 studies (N = 22,279). Using data from the discovery cohorts, single nucleotide polymorphisms (SNPs) at five independent loci reached genome-wide significance (*P* values < 5 × 10^–8^) and SNPs at another 22 loci showed suggestive associations with early-life head circumference (5 × 10^–8^ < *P* values < 5 × 10^−6^). A Manhattan plot of the discovery meta-analysis is shown in Fig. 1. No evidence of inflation by population stratification or cryptic relatedness was found (genomic inflation factor (λ) = 1.02 and LD-score regression intercept = 1.0) (Additional file 1: Fig. S1) [21]. The index SNPs from each of the 27 genome-wide and suggestive loci were followed up in four replication cohorts (N = 6913). The results of the discovery, replication and combined analyses are shown in Table 1 and Additional file 1: Tables S2 and S3. Results of the discovery analysis for SNPs with *P* values < 5 × 10^−6^ are shown in Additional file 3: Table S4.

**Fig. 2:**
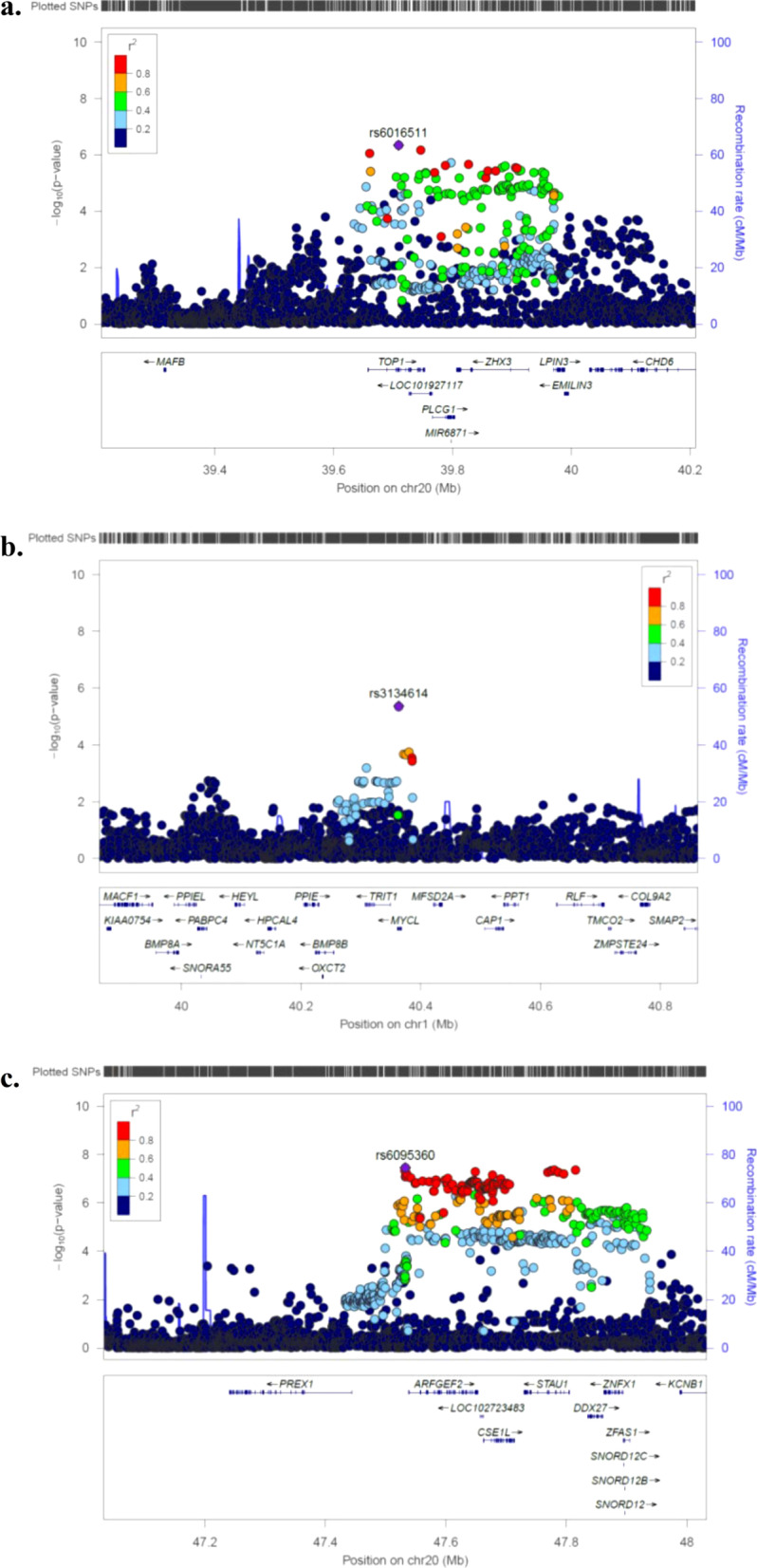
**a**–**c** Locus zoom plots of the 3 novel loci. Shown are the results of the meta-analysis. Regional association plot of the 3 novel loci. SNPs are plotted with their *P* values from the discovery stage (as − log10; left y-axis) as a function of genomic position (x-axis). Estimated recombination rates (right y-axis) taken from 1000 Genomes, March 2012 release are plotted to reflect the local LD-structure around the top associated SNP (indicated with purple color) and the correlated proxies (indicated in colors)

Of the 27 SNPs identified in the discovery meta-analysis, seven reached genome-wide significance in the combined meta-analysis, in which we used data from the discovery and replication stage. An identified locus was defined to be a known locus if the index SNP was within a range of 500 kb upstream to 500 kb downstream of and in LD (r^2^ ≥ 0.2) with a previously reported SNP for head circumference, intracranial volume, or brain volume at any age [7–11]. Of the seven genome-wide significant SNPs, three were novel: rs6095360 near *ARFGEF2*, rs3134614 near *MYCL1*, and rs6016511 near *TOP1* (Table 1 and Additional file 1: Tables S2 and S3). Regional plots of these three loci are shown in Fig. 2. The remaining four SNPs mapped to loci previously identified from GWAS on infant head circumference, adult intracranial volume, and/or adult brain volume (nearest genes: *HMGA2*, *C12orf65*, *NT5C2*, and *GRB10*) [7, 10, 11].

Six SNPs located within 500 kb (upstream or downstream) from rs6095360 (*ARFGEF2*), rs3134614 (*MYCL1*), and rs6016511 (*TOP1*) have been previously reported in relation to adult height [30]. The linkage disequilibrium (LD) of the three novel SNPs near *ARFGEF2*, *MYCL1*, and *TOP1* with these six adult height SNPs was weak to moderate. We found suggestive evidence of association for rs6095360 (*ARFGEF2*) with early-life length in 28,949 participants between 6 and 30 months of age in an unpublished GWAS meta-analysis of 24 cohorts (*P* value 4.58 × 10^–7^), but the other two novel SNPs did not show evidence of association (Additional file 1: Table S5).

We also performed a meta-analysis of birth head circumference in a total of 32,084 participants. None of the SNPs reached genome-wide significance in this analysis. A total of 11 SNPs with *P* values between 5 × 10^–8^ and 5 × 10^–6^ were taken forward for replication (N = 3750) and combined analyses, but none were genome-wide significant in the combined analysis. Therefore, no follow-up analyses were performed for birth head circumference. A Manhattan plot and a Quantile–Quantile plot of the discovery meta-analysis of birth head circumference are shown in Additional file 1: Figs. S2 and S3**.** Results of the discovery analysis for SNPs with *P* values < 5 × 10^−6^ are shown in Additional file 4: Table S6.

### Functional characterization

To gain insight into the function of the seven SNPs associated with early-life head circumference, we used several strategies. First, using Bayesian colocalization analysis, we examined evidence of colocalization between GWAS and eQTL signals for the seven index SNPs (GTEx v7), but did not find a signal at any of the seven loci. Second, to explore biological processes, we used the Kyoto Encyclopedia of Genes and Genomes (KEGG) database in the Database for Annotation, Visualization and Integrated Discovery (DAVID) with the seven SNPs and their nearest genes as input [27, 28], but no enriched biological processes were identified. Third, we did a look-up of the seven nearest genes in mouse-knockout data but there was no phenotypic information available for any of these gene knockouts [31]. Fourth, we examined gene expression profiles for the nearest genes to the seven SNPs with GTEx v7 in 53 tissues, using the tool for Functional Mapping and Annotation of Genome-Wide Association Studies (FUMA) [24, 25]. We did not find significant differential expression for these seven nearest genes. Going a step further, we included all genes within a range of 500 kb upstream to 500 kb downstream of the seven index SNPs and found significant differential expression in several brain structures, including the putamen, amygdala, hippocampus, caudate, nucleus accumbens, substantia nigra, and anterior cingulate cortex, and in other tissues such as the heart, pancreas and liver [25].

### Shared genetic background of early-life head circumference with childhood and adult outcomes

**Fig. 3:**
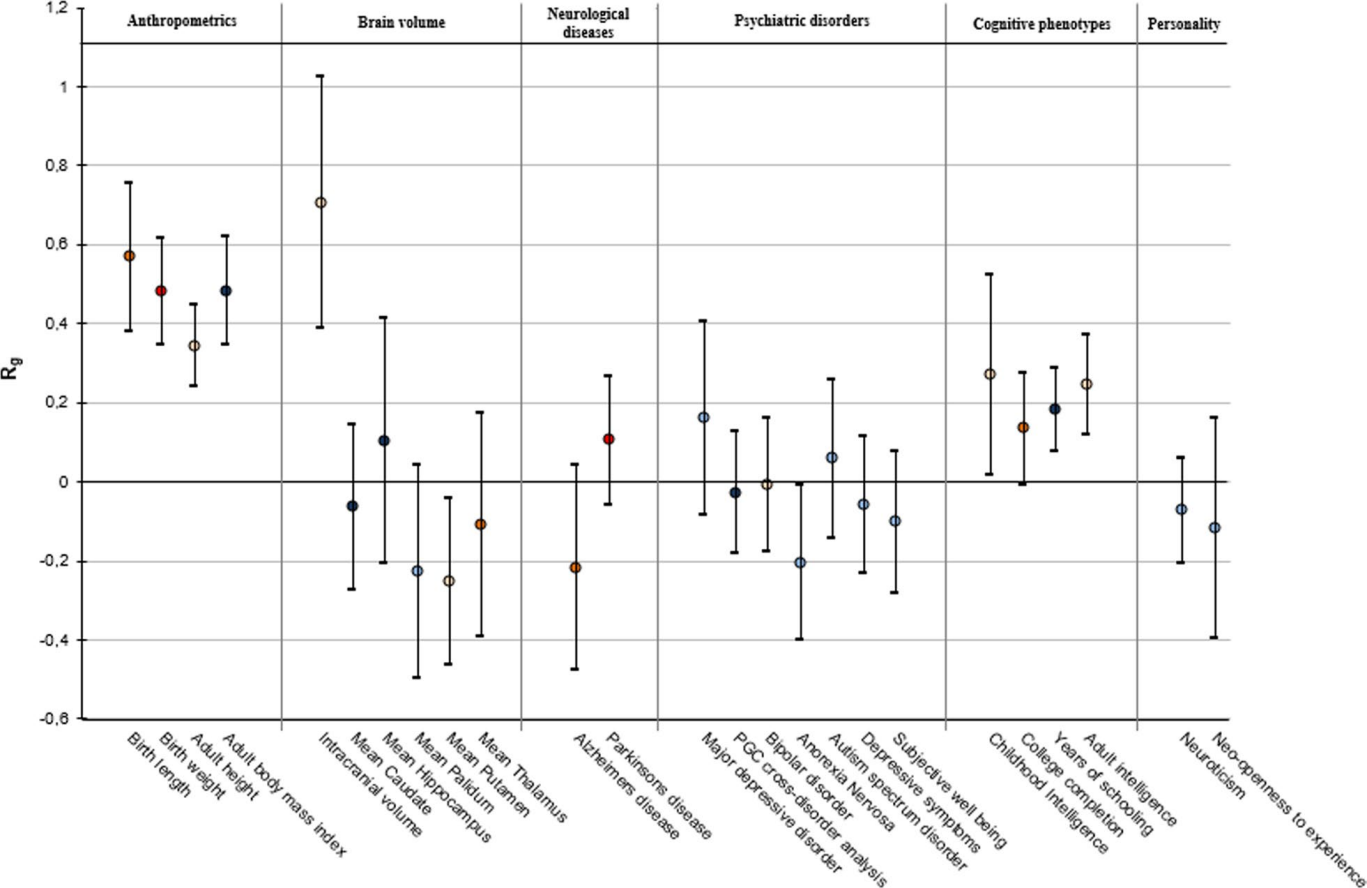
Genome-wide genetic correlations between early-life head circumference and adult traits and diseases. On the x axis the traits and diseases are shown. The y-axis shows the genetic correlations (R_g_) and corresponding standard errors, indicated by error bars, between early-life head circumference and each trait, estimated by LD score regression. The genetic correlation estimates (R_g_) are colored according to their intensity and direction. Red indicates a positive correlation, blue indicates a negative correlation. References can be found in Additional file 1: Table S7

First, to examine whether associations between early-life head circumference and phenotypes in later life from observational studies were at least partly genetically explained, we estimated genetic correlations between early-life head circumference and other traits, using LD score regression, which is based on the genome-wide meta-analysis results [29]. We found positive genetic correlation coefficients for early-life head circumference with birth length (R_g_ = 0.57, *P* value = 3.61 × 10^–12^), birth weight (R_g_ = 0.48, *P* value = 2.51 × 10^–8^), adult height (R_g_ = 0.34, *P* value = 9.77 × 10^–11^), adult body mass index (BMI) (R_g_ = 0.13, *P* value = 0.03), adult intracranial volume (R_g_ = 0.71, *P* value = 1.44 × 10^–5^) and several cognitive outcomes, including childhood intelligence (age range 6–18 years) (R_g_ = 0.27, *P* value = 0.04), years of schooling (R_g_ = 0.18, *P* value = 6.0 × 10^–4^), and adult intelligence (R_g_ = 0.25, *P* value = 1.0 × 10^–4^), (Fig. 3 and Additional file 1: Table S7). We did not find genetic correlations with any psychiatric (for instance bipolar disorder and autism spectrum disorder), neurological (Parkinson’s disease, Alzheimer’s disease) or personality-related outcomes (for instance neuroticism) (*P* values > 0.05) (Additional file 1: Table S7).

Second, we performed a look-up of the seven identified SNPs in GWAS meta-analyses data on potentially related phenotypes, including adult intracranial volume, intelligence, Alzheimer’s disease, neuroticism, depression and educational attainment [7, 32–35]. Rs10883848 (*NT5C2*) was genome-wide significantly associated with adult intracranial volume (*P* value = 1.48 × 10^–9^). All effect estimates for intracranial volume were in the same direction as those for head circumference in early-life (Additional file 1: Table S8). Additionally, rs6095360 (*ARFGEF2*) was associated with adult intelligence at a genome-wide level (*P* value = 2.31 × 10^–16^). We did not observe evidence of genome-wide significant associations with Alzheimer’s disease, neuroticism, depression or educational attainment. Suggestive evidence for association was observed for rs8756 (*HMGA2*) with adult intracranial volume (*P* value = 6.32 × 10^–8^), for rs9795522 (*C12orf65*) with adult intelligence (*P* value = 1.41 × 10^–6^) and for rs8756 (*HMGA2*) with educational attainment (*P* value = 1.41 × 10^–6^) (Additional file 1: Tables S9–13). None of the SNPs showed evidence of association with birth head circumference in the GWAS meta-analysis (Additional file 1: Table S14).

**Fig. 4:**
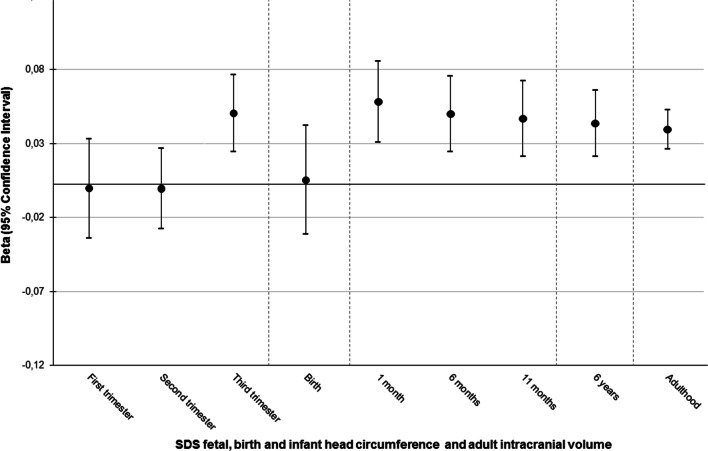
Associations of early-life head circumference genetic risk score with head circumference at different time points in the Generation R Study and from UK Biobank data. On the x axis the different ages are shown at which the genetic risk score of the seven early-life head circumference SNPs is tested. On the y axis the beta’s and 95% confidence intervals from linear regression analyses are shown. Detailed data can be found in Additional file 1: Table S15

Third, we calculated a combined genetic risk score (GRS) using the seven index SNPs identified in the current study. We summed the number of head circumference-increasing alleles weighted by the effect sizes from the combined meta-analysis after excluding the Generation R Study, in which we tested the GRS longitudinally. The GRS was associated with fetal head circumference in the third trimester of pregnancy (N = 1984), at postnatal ages 1 month (N = 1501), 6 months (N = 1662), 11 months (N = 1528), and 6 years (N = 4010) and at the mean (SD) age of 64 (7.5) years (N = 22,152) in UK Biobank data (*P* values < 0.05) (Fig. 4 and Additional file 1: Table S15).

## Discussion

In a GWAS meta-analysis including 29,192 participants of European ancestry aged 6 to 30 months of age, we identified seven genome-wide significant SNPs associated with early-life head circumference, of which three were novel and had not been related with head circumference, intracranial volume or brain volume before. We observed positive genetic correlations between early-life head circumference and adult intracranial volume as well as cognitive outcomes.

We used multiple approaches to identify potential underlying mechanisms. As there is no strong evidence linking the nearest genes to the seven SNPs as causal genes, we included all genes within 500 kb to either side of the genome-wide significant SNPs in a GTEx analysis. We found differential expression of these genes in different brain structures that are related to cognitive functions and emotional control, indicating a potential functional role of these genes in the brain [36–39]. However, as donors aged 20–79 years were included in the GTEx data source, we were not able to look at expression of the genes in brain structures in early life. Using colocalization analysis, no potentially causal genes were identified [26]. Future studies should also determine whether the nearest genes, identified in this study, are indeed the causal genes and assess their expression in child brain structures.

The potential roles of the nearest genes to the novel loci are still poorly understood. *MYCL1*, (MYCL proto-oncogene, BHLH transcription factor), and *TOP1* (DNA topoisomerase 1) have been suggested to play a role in various types of cancer [40–44]. The role of these genes in the development of head circumference in early life is currently unknown. A mutation in *ARFGEF2* has been previously associated with several phenotypes related to brain development, including microcephaly [45, 46]. The three novel SNPs are located near regions that have been previously reported for adult height, indicating that they might represent loci involved in growth [30]. However, the strong association of rs6095360 (nearest gene: *ARFGEF2*) with adult intelligence (*P* value = 2.31 × 10^–16^) might indicate a role in brain development as well [32]. Future functional studies should investigate the role of these genes and should determine whether these genes are indeed causal.

Observational studies suggest that early life head circumference is not only related to intracranial volume in adults, but also to adult intelligence, Alzheimer’s disease, schizophrenia and autism [1–3, 12–17]. In observational studies of such associations, effect estimates may be influenced by confounding factors and reverse causation, potentially evoking spurious associations [47, 48]. Genetic studies such as ours can provide more insight into the etiology of complex diseases. We found a strong genetic correlation of head circumference in early life with intracranial volume in adults, underlining the idea that early-life head circumference is a valid measure for brain growth during early development [3, 7]. Abnormal growth trajectories of head circumference are related to adverse neurological outcomes [49]. Additionally, variation within the normal range of head circumference has been reported to be associated with cognitive and behavioral traits [2, 50, 51]. In the current study, we observed positive genetic correlations for head circumference in early life with childhood intelligence, years of schooling and adult intelligence. These findings indicate that the association of early-life head circumference with cognitive function from observational studies is at least partly explained by a shared genetic background, which is in line with the observed positive genetic correlations between intracranial volume and cognitive function in the literature [7, 8, 52]. Altogether, the findings from the current study and from previous literature suggest that the associations between measures of early-life brain volume and cognition decades later are partly genetically explained.

It has been suggested that heritability estimates are consistent from childhood onwards [6]. Whether this genetic stability starts from early life onwards, is currently not well studied. We observed evidence for association of two of the seven SNPs with adult intracranial volume [7]. We combined the seven index SNPs into a weighted GRS. Although we have used effect sizes of the meta-analysis after excluding the Generation R Study, the discovery of the seven SNPs was based on the meta-analysis including the Generation R Study, potentially resulting in overfitting of the GRS. We found an association of the GRS with fetal head circumference in third trimester, head circumference in infancy and childhood and intracranial volume in adulthood. The effect estimates were largely similar for the different time points. We did not observe an association of the GRS with birth head circumference. This may be explained by the larger variance in birth head circumference that might be present due to the deformation of the head during birth. Also, in the GWAS meta-analysis of head circumference at birth, no SNPs were genome-wide significantly associated with birth head circumference. Thus, the genetic background of early-life head circumference seems to partially overlap with the genetic background of related measures in later life. The SNPs identified in infancy seem to represent effects across multiple ages. However, as not all SNPs identified for early-life head circumference were associated with adult intracranial volume, it has been suggested that some of the underlying mechanisms are age-specific.

## Conclusions

We identified seven SNPs associated with early-life head circumference. Three of these are novel and four mapped to loci that are known for head circumference, intracranial volume, or brain volume. We observed a strong positive genetic correlation of early-life head circumference with adult intracranial volume and cognitive outcomes in childhood and adulthood. The well-known associations of head circumference with later cognitive phenotypes are partly explained by genetics. Our findings may contribute to the understanding of the early-life brain development, which may lay the foundation for diseases later in life.

## Supplementary Information

**Additional file 1**. Additional data includes 15 tables, 3 figures and additional cohort information and methods.

**Additional file 2.** Characteristics of discovery and replication studies.

**Additional file 3.** Results of the discovery analysis for SNPs with P-values <5 × 10−6.

**Additional file 4.** Results of the discovery analysis for SNPs with P-values <5 × 10−6 for birth head circumference.

## Data Availability

GWAS summary data will be deposited at the EGG website (https://egg-consortium.org/) at publication. Individual study data are available from the corresponding author on reasonable request.

## Abbreviations

GWAS: Genome-wide association studies
SDS: Standard deviation scores
MAF: Minor allele frequency
KEGG: Kyoto Encyclopedia of Genes and Genomes
HRC: Haplotype Reference Consortium
SNPs: Single nucleotide polymorphisms
LD: Linkage disequilibrium
DAVID: Database for Annotation, Visualization and Integrated Discovery
FUMA: Functional Mapping and Annotation of Genome-Wide Association Studies
BMI: Body mass index
GRS: Genetic risk score
