# Supplementary material for: Genetics of early-life head circumference and genetic correlations with neurological, psychiatric and cognitive outcomes
Source: BMC Med Genomics. 2022 Jun 4;15:124. doi: 10.1186/s12920-022-01281-1 (PMC9166310; doi:10.1186/s12920-022-01281-1)
Supplement: Supplementary file 1 — Additional file 1. Additional data includes 15 tables, 3 figures and additional cohort information and methods. [file 12920_2022_1281_MOESM1_ESM.docx]

**Supplementary Information**

**Genetics of early-life head circumference and genetic correlations with neurological, psychiatric and cognitive outcomes**

Suzanne Vogelezang, ^1-3^, Jonathan P Bradfield^4,5,^  the Early Growth Genetics Consortium*, Struan FA Grant^4,59,110-111^, Janine F Felix^1,2^, Vincent WV Jaddoe^1,2^

1 The Generation R Study Group, Erasmus MC, University Medical Center Rotterdam, the Netherlands.

2 Department of Pediatrics, Erasmus MC, University Medical Center Rotterdam, the Netherlands.

3 Department of Epidemiology, Erasmus MC, University Medical Center Rotterdam, the Netherlands.

4 Center for Applied Genomics, Division of Human Genetics, Children’s Hospital of Philadelphia, Philadelphia, PA, USA.

5 Quantinuum Research LLC, San Diego, CA, USA.

110 Division of Endocrinology and Diabetes, The Children’s Hospital of Philadelphia, Philadelphia, PA, USA.

111 Center for Spatial and Functional Genomics, Division of Human Genetics, Children’s Hospital of Philadelphia, Philadelphia, PA, USA.

* A list of authors and their affiliations appears at the end of the paper.

These authors contributed equally: Suzanne Vogelezang and Jonathan P Bradfield.
These authors contributed equally: Struan FA Grant, Janine F Felix, Vincent WV Jaddoe.

# Corresponding author: [v.jaddoe@erasmusmc.nl](mailto:v.jaddoe@erasmusmc.nl)

**Fig S1** Quantile-Quantile plot of the SNPs in the discovery meta-analysis of early-life head circumference in 21 studies

**
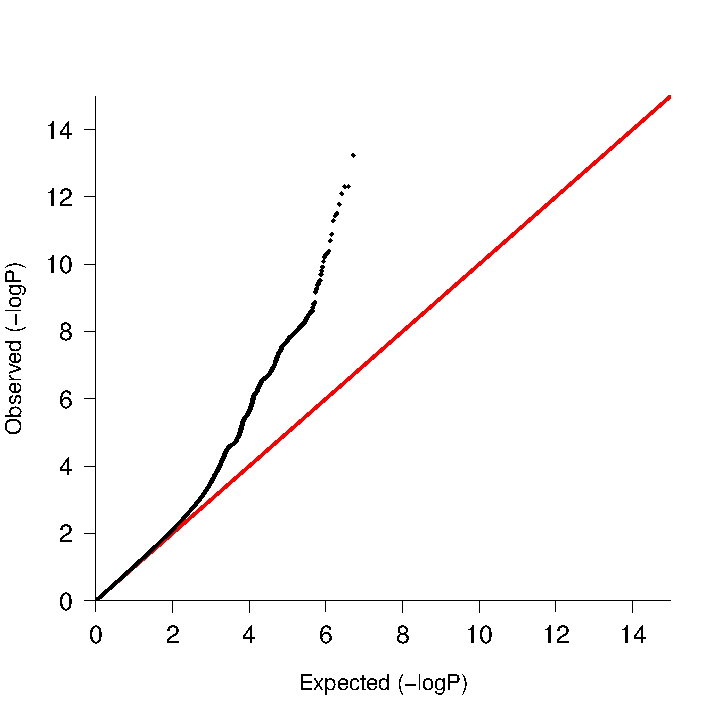
**

**λ= 1.02**

**Fig S2** Manhattan plot of results of the discovery meta-analysis of birth head circumference in 22 studies


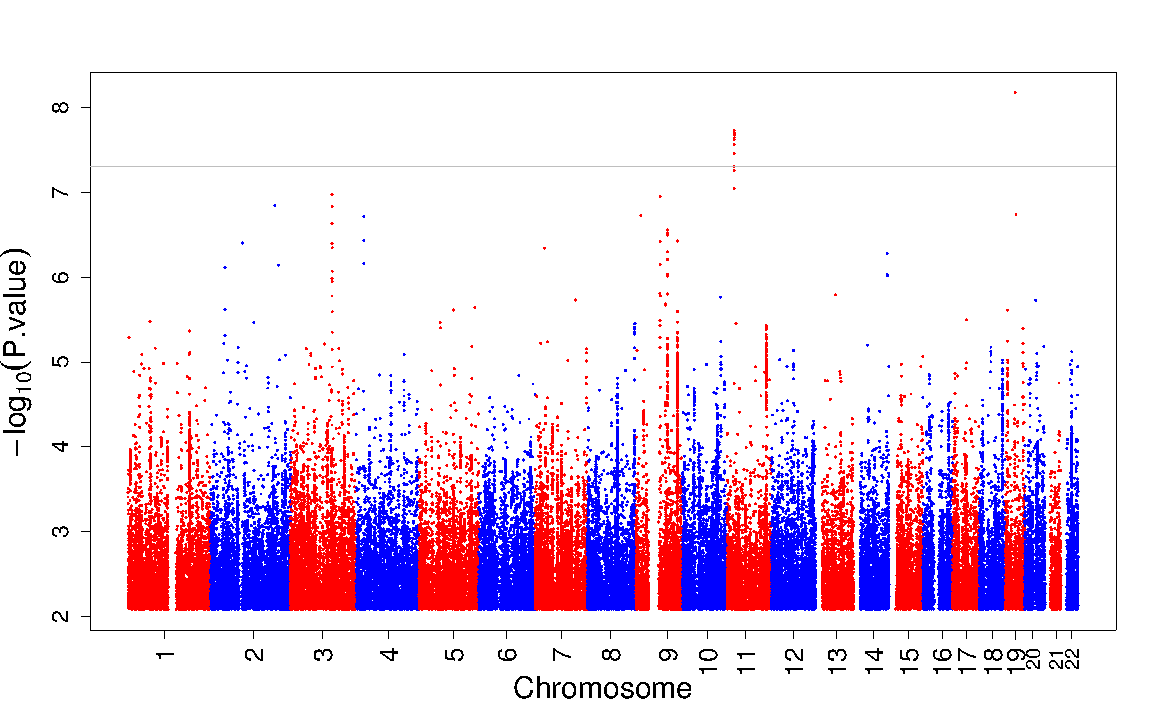


**Fig S3** Quantile-Quantile plot of the SNPs in the discovery meta-analysis of birth head circumference in 22 studies

**
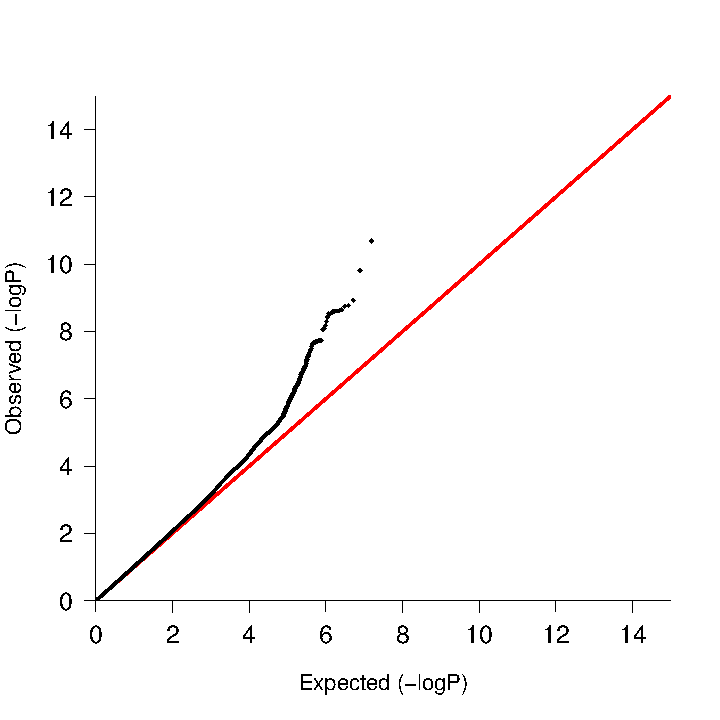
**

**λ= 1.02**

**Table S1** Descriptive characteristics of studies

*See separate Excel spreadsheet*

**Table S2** Results of the discovery, replication and combined analyses for all 27 SNPs with *P-*values <5 x 10^-6^ in the discovery analysis of early-life head circumference

|  | | | | | | | **Discovery analysis** | | | | **Replication analysis** | | | **Combined analysis** | | |
| --- | --- | --- | --- | --- | --- | --- | --- | --- | --- | --- | --- | --- | --- | --- | --- | --- |
| **SNP** | **CHR** | **Position** | **Nearest gene** | **EA/**  **Non-EA** | **EAF^a^** | | **Beta** | **SE** | **P-value** | **Hetero-geneity**  **I^2^** | **Beta** | **SE** | ***P*-value** | **Beta** | **SE** | **P-value** |
| rs8756^b, c, d^ | 12 | 66359752 | *HMGA2* | C/A | 0.50 | | 0.071 | 0.010 | 5.18 x 10^-12^ | 0 | 0.049 | 0.017 | 4.62 x 10^-3^ | 0.065 | 0.009 | **1.84 x 10^-13^** |
| rs9795522^b^ | 12 | 123730935 | *C12orf65* | C/A | 0.23 | | 0.072 | 0.012 | 3.20 x 10^-9^ | 0 | 0.040 | 0.018 | 6.19 x 10^-4^ | 0.072 | 0.010 | **7.37 x 10^-12^** |
| rs10883848^d^ | 10 | 104973061 | *NT5C2* | A/G | 0.35 | | 0.052 | 0.011 | 3.06 x 10^-6^ | 51.9 | 0.075 | 0.018 | 3.86 x 10^-5^ | 0.058 | 0.010 | **9.99 x 10^-10^** |
| rs6095360 | 20 | 47532536 | *ARFGEF2* | G/A | 0.32 | | 0.061 | 0.011 | 3.53 x 10^-8^ | 0 | 0.040 | 0.018 | 0.03 | 0.055 | 0.009 | **4.39x 10^-9^** |
| rs3134614 | 1 | 40363054 | *MYCL1* | C/G | 0.88 | | 0.072 | 0.016 | 4.31 x 10^-6^ | 18.3 | 0.086 | 0.026 | 7.75 x 10^-4^ | 0.075 | 0.013 | **1.43 x 10^-8^** |
| rs6016511 | 20 | 39709255 | *TOP1* | T/C | 0.68 | | 0.055 | 0.011 | 4.55 x 10^-7^ | 16.1 | 0.042 | 0.019 | 0.02 | 0.052 | 0.010 | **4.02 x 10^-8^** |
| rs7792211^d^ | 7 | 50707141 | *GRB10* | G/A | 0.42 | | 0.055 | 0.011 | 7.62 x 10^-7^ | 0 | 0.042 | 0.017 | 0.01 | 0.051 | 0.009 | **4.43 x 10^-8^** |
| rs1490384^c,d^ | 6 | 126851160 | *CENPW* | T/C | 0.51 | | 0.058 | 0.011 | 5.46 x 10^-8^ | 0 | 0.026 | 0.017 | 0.14 | 0.049 | 0.009 | 5.70 x 10^-8^ |
| rs116536930 | 17 | 43855156 | *CRHR1* | G/T | 0.51 | | 0.061 | 0.011 | 4.19 x 10^-8^ | 0 | 0.027 | 0.043 | 0.53 | 0.055 | 0.011 | 2.27 x 10^-7^ |
| rs72952297 | 2 | 180379814 | *ZNF385B* | T/G | 0.93 | | 0.098 | 0.021 | 2.07 x 10^-6^ | 27.6 | 0.057 | 0.035 | 0.11 | 0.087 | 0.018 | 9.19 x 10^-7^ |
| rs12350281 | 9 | 20637773 | *MLLT3* | G/A | 0.05 | | 0.145 | 0.031 | 4.22 x 10^-6^ | 0 | 0.396 | 0.187 | 0.03 | 0.152 | 0.031 | 9.98 x 10^-7^ |
| rs7658192 | 4 | 140023559 | *ELF2* | T/G | 0.33 | | 0.058 | 0.011 | 2.29 x 10^-7^ | 15.3 | 0.014 | 0.019 | 0.46 | 0.046 | 0.010 | 1.70 x 10^-6^ |
| rs143094271^e^ | 17 | 7463102 | *TNFSF12-TNFSF13* | A/G | 0.02 | | 0.160 | 0.032 | 5.96 x 10^-7^ | 0 | 0.045 | 0.058 | 0.44 | 0.133 | 0.028 | 2.02 x 10^-6^ |
| rs1014098 | 1 | 119458215 | *TBX15* | C/T | 0.79 | | 0.059 | 0.013 | 2.67 x 10^-6^ | 0 | 0.029 | 0.021 | 0.17 | 0.051 | 0.011 | 2.05 x 10^-6^ |
| rs77690628 | 8 | 26189223 | *PPP2R2A* | A/T | 0.07 | | 0.100 | 0.021 | 1.02 x 10^-6^ | 0 | 0.032 | 0.032 | 0.31 | 0.080 | 0.017 | 3.26 x 10^-6^ |
| rs9853018 | 3 | 141101961 | *ZBTB38* | T/C | 0.45 | | 0.051 | 0.010 | 5.41 x 10^-7^ | 24.9 | 0.009 | 0.017 | 0.62 | 0.040 | 0.009 | 5.30 x 10^-6^ |
| rs4674101 | 2 | 217487622 | *RPL37A* | A/G | 0.31 | | 0.055 | 0.011 | 8.66 x 10^-7^ | 6.9 | 0.010 | 0.019 | 0.57 | 0.043 | 0.010 | 6.56 x 10^-6^ |
| rs150266910 | 1 | 23442265 | *LUZP1* | C/T | 0.83 | | 0.066 | 0.014 | 1.42 x 10^-6^ | 10.4 | 0.011 | 0.023 | 0.65 | 0.052 | 0.012 | 1.04 x 10^-5^ |
| rs13067734 | 3 | 141963015 | *GK5* | A/C | 0.52 | | 0.047 | 0.010 | 2.83 x 10^-6^ | 0.9 | 0.010 | 0.017 | 0.55 | 0.037 | 0.009 | 1.22 x 10^-5^ |
| rs17223831 | 14 | 90601920 | *KCNK13* | A/G | 0.14 | | 0.077 | 0.016 | 1.24 x 10^-6^ | 21.5 | -0.082 | 0.060 | 0.18 | 0.067 | 0.015 | 1.30 x 10^-5^ |
| rs62580922 | 9 | 125631594 | *RC3H2* | C/T | 0.07 | | 0.112 | 0.020 | 1.13 x 10^-8^ | 0 | -0.030 | 0.031 | 0.35 | 0.072 | 0.017 | 1.52 x 10^-5^ |
| rs2448415 | 1 | 93469310 | *FAM69A* | A/G | 0.47 | | 0.049 | 0.010 | 1.97 x 10^-6^ | 36.5 | 0.004 | 0.017 | 0.81 | 0.037 | 0.009 | 2.56 x 10^-5^ |
| rs261752 | 5 | 39328506 | *C9* | A/G | 0.55 | 0.049 | | 0.010 | 1.39 x 10^-6^ | 0 | -0.007 | 0.017 | 0.70 | 0.035 | 0.009 | 7.63 x 10^-5^ |
| rs72979897 | 6 | 140881181 | *MIR3668* | T/A | 0.04 | 0.140 | | 0.030 | 4.13 x 10^-6^ | 0 | 0.007 | 0.046 | 0.88 | 0.100 | 0.025 | 8.26 x 10^-5^ |
| rs503783 | 6 | 161959649 | *PARK2* | T/C | 0.27 | 0.054 | | 0.012 | 2.98 x 10^-6^ | 0 | -0.009 | 0.019 | 0.66 | 0.037 | 0.010 | 1.59 x 10^-4^ |
| rs113397574 | 5 | 31968320 | *PDZD2* | T/C | 0.02 | 0.171 | | 0.037 | 4.56 x 10^-6^ | 0 | -0.032 | 0.063 | 0.61 | 0.118 | 0.032 | 2.40 x 10^-4^ |
| rs77535478 | 9 | 77533287 | *TRPM6* | A/G | 0.04 | 0.169 | | 0.035 | 1.28 x 10^-6^ | 0 | -0.035 | 0.040 | 0.39 | 0.081 | 0.026 | 2.05 x 10^-3^ |

^a^ From combined analysis
^b^ Locus previously reported for infant head circumference

^c^ Locus previously reported for adult intracranial volume

^d^ Locus previously reported for adult head brain volume

^e^ Locus previously reported for childhood and adult head circumference and intracranial volume

CHR: Chromosome; EA: Effect Allele; EAF: Effect Allele Frequency; SE: Standard Error

**Table S3** Directions of effect for the individual discovery and replication studies for all 27 SNPs with *P*-values <5 x 10^-6^ in the discovery analysis of early-life head circumference

| **SNP** | **CHR** | **Position** | **Nearest gene** | **EA/**  **Non-EA** | **Direction of effect discovery analysis** | **Direction of effect replication analysis** | **P–value combined analysis** | **Heterogeneity I^2^ combined analysis** |
| --- | --- | --- | --- | --- | --- | --- | --- | --- |
| rs8756^a, b, c^ | 12 | 66359752 | *HMGA2* | C/A | +++++++?++??+++++?+++ | -+++- | **1.84 x 10^-13^** | 0 |
| rs9795522^a^ | 12 | 123730935 | *C12orf65* | C/A | ++++++++-+-++++++++++ | -++++ | **7.37 x 10^-12^** | 0 |
| rs10883848^c^ | 10 | 104973061 | *NT5C2* | A/G | +++--+++-+++?+++--++- | +++++ | **9.99 x 10^-10^** | 54.8 |
| rs6095360 | 20 | 47532536 | *ARFGEF2* | G/A | ?++++++-+--++++++++-+ | ++-++ | **4.39 x 10^-9^** | 36.6 |
| rs3134614 | 1 | 40363054 | *MYCL1* | C/G | -++++-++-+-+++++++++- | ++-++ | **1.43 x 10^-8^** | 0 |
| rs6016511 | 20 | 39709255 | *TOP1* | T/C | ?+-+++++++??+++++++-- | -++++ | **4.02 x 10^-8^** | 0 |
| rs7792211^d^ | 7 | 50707141 | *GRB10* | G/A | ++++--++-+--?++++-+++ | +++++ | **4.43 x 10^-8^** | 11.3 |
| rs1490384^c,d^ | 6 | 126851160 | *CENPW* | T/C | ++-+-?+?++??++++??+++ | -++++ | 5.70 x 10^-8^ | 43.9 |
| rs116536930 | 17 | 43855156 | *CRHR1* | G/T | ++++++++-+++?++++?++- | +?-+- | 2.27 x 10^-7^ | 40.1 |
| rs72952297 | 2 | 180379814 | *ZNF385B* | T/G | ?+-++-+-+--+++++++++- | +++++ | 9.19 x 10^-7^ | 0 |
| rs12350281 | 9 | 20637773 | *MLLT3* | G/A | ++-+++++++++?++++-+++ | ??++? | 9.98 x 10^-7^ | 43.1 |
| rs7658192 | 4 | 140023559 | *ELF2* | T/G | ++++-++++-++?++++-+++ | -+++- | 1.70 x 10^-6^ | 62.9 |
| rs143094271^d^ | 17 | 7463102 | *TNFSF12-TNFSF13* | A/G | ++--++++-+++++++-++++ | ++++? | 2.02 x 10^-6^ | 33.7 |
| rs1014098 | 1 | 119458215 | *TBX15* | C/T | +-++++++++-++++-+++++ | ++++- | 2.05 x 10^-6^ | 30.3 |
| rs77690628 | 8 | 26189223 | *PPP2R2A* | A/T | ?++++++-++-++++-+++-+ | +++++ | 3.26 x 10^-6^ | 24.6 |
| rs9853018 | 3 | 141101961 | *ZBTB38* | T/C | ?+-+++++-++++++-+++-+ | ++-+- | 5.30 x 10^-6^ | 38.4 |
| rs4674101 | 2 | 217487622 | *RPL37A* | A/G | ++-+++++++++++++-+-+- | ++-+- | 6.56 x 10^-6^ | 65.3 |
| rs150266910 | 1 | 23442265 | *LUZP1* | C/T | ++++-++-++++?+++--++- | +-+++ | 1.04 x 10^-5^ | 58.2 |
| rs13067734 | 3 | 141963015 | *GK5* | A/C | ++-+++++++++++++--+-+ | ++-++ | 1.22 x 10^-5^ | 26.5 |
| rs17223831 | 14 | 90601920 | *KCNK13* | A/G | -+++-+++++++?+++-++-+ | -?-+- | 1.30 x 10^-5^ | 67.6 |
| rs62580922 | 9 | 125631594 | *RC3H2* | C/T | ?++++++++++++++++++++ | +--+- | 1.52 x 10^-5^ | 77.8 |
| rs2448415 | 1 | 93469310 | *FAM69A* | A/G | ?+++-++++++-+++-+++-- | -+-++ | 2.56 x 10^-5^ | 60.7 |
| rs261752 | 5 | 39328506 | *C9* | A/G | ?++++++++++-++++++-+- | --+++ | 7.63 x 10^-5^ | 63.7 |
| rs72979897 | 6 | 140881181 | *MIR3668* | T/A | ?--++-+--++++++++++++ | -+-++ | 8.26 x 10^-5^ | 50.6 |
| rs503783 | 6 | 161959649 | *PARK2* | T/C | ++++++++++++++-++++++ | -+++- | 1.59 x 10^-4^ | 74.3 |
| rs113397574 | 5 | 31968320 | *PDZD2* | T/C | ++++-+++++++-+++-++-+ | --++- | 2.40 x 10^-4^ | 58.5 |
| rs77535478 | 9 | 77533287 | *TRPM6* | A/G | ?++-++++++-+?++++++++ | --+++ | 2.05 x 10^-3^ | 75.4 |

CHR, chromosome; EA, effect allele; EAF, effect allele frequency.
Bolded *P*-values indicate genome-wide significance in the combined analysis.

^a^ Locus previously reported for infant head circumference

^b^ Locus previously reported for adult intracranial volume

^c^ Locus previously reported for adult head brain volume

^d^ Locus previously reported for childhood and adult head circumference and intracranial volume

Direction of the effect for the effect allele for each individual study is shown: + indicates a positive effect estimate for the effect allele, - indicates a negative effect estimate for the effect allele, ? indicates no information available. Order of the studies in the discovery analysis: INMAMEN, SAB, VAL, The Generation R Study, COPSAC 2000, COPSAC 2010, CHOP Philadelphia, ABCD, ALSPAC, DNBC GOYA-offspring, TEENAGE, STRIP, SKOT 2, SKOT 1, Raine Study, NFBC 1966, GINILISA, DNBC-PTB, PANIC, NFBC 1986, LIFE-Child, MOBA term, MOBA preterm. Order of the studies in the replication analysis: EFSOCH, MOBA, CHOP Europe, INMA GIP.

**Table S4** Results of the discovery analysis of early-life head circumference for all SNPs with *P*-values <5 x 10^-6^- *Separate Excel sheet*

**Table S5** Associations of seven early-life head circumference SNPs (*P-*values <5 × 10^-8^) with early-life length

| **SNP** | **CHR** | **Position** | **Nearest gene** | **EA/non_EA** | **EAF^a^** | **Beta^a^** | **SE^a^** | **P-value ^a^** | **N^a^** |
| --- | --- | --- | --- | --- | --- | --- | --- | --- | --- |
| rs8756 | 12 | 66359752 | *HMGA2* | C/A | 0.48 | 0.062 | 0.010 | **1.50 x 10^-10^** | 25619 |
| rs9795522 | 12 | 123730935 | *C12orf65* | C/A | 0.23 | 0.043 | 0.011 | 9.08 x 10^-5^ | 28949 |
| rs10883848 | 10 | 104973061 | *NT5C2* | A/G | 0.35 | 0.018 | 0.010 | 0.07 | 27587 |
| rs6095360 | 20 | 47532536 | *ARFGEF2* | G/A | 0.31 | 0.050 | 0.010 | 4.58 x 10^-7^ | 28011 |
| rs3134614 | 1 | 40363054 | *MYCL1* | C/G | 0.88 | 0.012 | 0.014 | 0.41 | 28949 |
| rs6016511 | 20 | 39709255 | *TOP1* | T/C | 0.68 | 0.006 | 0.010 | 0.54 | 26516 |
| rs7792211 | 7 | 50707141 | *GRB10* | G/A | 0.41 | 0.008 | 0.010 | 0.45 | 27587 |

CHR, chromosome; EA, effect allele; EAF, effect allele frequency; SE, standard error.
Bolded *P*-values indicate genome-wide significance.
^a^ From early-life length analysis (unpublished data)
P*-*values < 5 × 10^-8^ are considered statistically significant.

**Table S6** Results of the discovery analysis of birth head circumference for all SNPs with *P*-values <5 x 10^-6^- *Separate Excel sheet*

**Table S7** Summary results of LD score regression analyses between early-life head circumference and various anthropometric, cognitive and psychiatric traits

| **Phenotype 1** | **Phenotype 2** | **Genetic correlation (R_g_)** | **SE** | **Z** | **P-value** | **Reference of**  **phenotype 2 (PMID)** |
| --- | --- | --- | --- | --- | --- | --- |
| Early-life head circumference | Birth length | 0.569 | 0.096 | 5901 | **3.61 x 10^-12^** | 25281659 |
| Early-life head circumference | Birth weight | 0.483 | 0.069 | 7.00 | **2.51 x 10^-8^** | 27680694 |
| Early-life head circumference | Childhood obesity | 0.129 | 0.080 | 16.12 | 0.11 | 22484627 |
| Early-life head circumference | Adult BMI | 0.131 | 0.058 | 22.40 | **0.03** | 20935630 |
| Early-life head circumference | Adult height | 0.344 | 0.053 | 64.70 | **9.77 x 10^-11^** | 20881960 |
| Early-life head circumference | Adult hip circumference | 0.206 | 0.053 | 36.69 | **2.0 x 10^-4^** | 25673412 |
| Early-life head circumference | Adult waist circumference | 0.127 | 0.054 | 23.78 | **0.02** | 25673412 |
| Early-life head circumference | Adult waist to hip ratio | -0.067 | 0.062 | -10.76 | 0.28 | 25673412 |
| Early-life head circumference | Intracranial volume | 0.706 | 0.163 | 43.38 | **1.44 x 10^-5^** | 25607358 |
| Early-life head circumference | Mean Caudate | -0.064 | 0.106 | -0.61 | 0.54 | 25607358 |
| Early-life head circumference | Mean Hippocampus | 0.102 | 0.158 | 0.65 | 0.52 | 25607358 |
| Early-life head circumference | Mean Pallidum | -0.227 | 0.138 | -16.44 | 0.10 | 25607358 |
| Early-life head circumference | Mean Putamen | -0.253 | 0.107 | -23.55 | **0.02** | 25607358 |
| Early-life head circumference | Mean Thalamus | -0.108 | 0.144 | -0.75 | 0.45 | 25607358 |
| Early-life head circumference | Years of schooling | 0.184 | 0.054 | 34.18 | **6.0 x 10^-4^** | 27225129 |
| Early-life head circumference | College completion | 0.134 | 0.073 | 18.36 | 0.07 | 23722424 |
| Early-life head circumference | Childhood IQ | 0.271 | 0.129 | 20.99 | **0.04** | 23358156 |
| Early-life head circumference | Adult intelligence | 0.246 | 0.064 | 3.83 | **1.0 x 10^-4^** | 28530673 |
| Early-life head circumference | Alzheimer’s disease | -0.218 | 0.132 | -16.50 | 0.10 | 24162737 |
| Early-life head circumference | Parkinson’s disease | 0.104 | 0.083 | 12.60 | 0.21 | 19915575 |
| Early-life head circumference | Neuroticism | -0.073 | 0.068 | -10.70 | 0.28 | 27089181 |
| Early-life head circumference | Neo-openness to experience | -0.118 | 0.142 | -0.83 | 0.41 | 21173776 |
| Early-life head circumference | Depressive symptoms | -0.058 | 0.088 | -0.665 | 0.51 | 27089181 |
| Early-life head circumference | Anorexia Nervosa | -0.205 | 0.100 | -20.49 | **0.04** | 24514567 |
| Early-life head circumference | Bipolar disorder | -0.006 | 0.086 | -0.07 | 0.94 | 21926972 |
| Early-life ead circumference | PGC cross-disorder analysis | -0.028 | 0.079 | -0.35 | 0.72 | 23453885 |
| Early-life head circumference | Major depressive disorder | 0.162 | 0.124 | 13.01 | 0.19 | 22472876 |
| Early-life head circumference | Autism spectrum disorder | 0.058 | 0.103 | 0.57 | 0.57 | - |
| Early-life head circumference | Subjective well-being | -0.102 | 0.092 | -11.14 | 0.27 | 27089181 |

SE: Standard Error

**Table S8** Associations of seven early-life head circumference SNPs (*P-*values <5 × 10^-8^) with adult intracranial volume

| **SNP** | **CHR** | **Position** | **Nearest gene** | **EA/non_EA** | **EAF^a^** | **Z-score^a^** | **P-value^a^** | **N^a^** |
| --- | --- | --- | --- | --- | --- | --- | --- | --- |
| rs8756 | 12 | 66359752 | *HMGA2* | C/A | 0.49 | 5.41 | 6.32 x 10^-8^ | 26577 |
| rs9795522 | 12 | 123730935 | *C12orf65* | C/A | 0.23 | 2.12 | 0.03 | 26577 |
| rs10883848 | 10 | 104973061 | *NT5C2* | A/G | 0.34 | 6.05 | **1.48 x 10^-9^** | 26577 |
| rs6095360 | 20 | 47532536 | *ARFGEF2* | G/A | 0.32 | 0.60 | 0.54 | 26577 |
| rs3134614 | 1 | 40363054 | *MYCL1* | C/G | 0.88 | 2.94 | 3.32 x 10^-3^ | 26577 |
| rs6016511 | 20 | 39709255 | *TOP1* | T/C | 0.68 | 2.27 | 0.02 | 26577 |
| rs7792211 | 7 | 50707141 | *GRB10* | G/A | 0.40 | 3.71 | 2.10 x 10^-4^ | 26577 |

CHR, chromosome; EA, effect allele; EAF, effect allele frequency.
Bolded *P*-values indicate genome-wide significance.
^a^ From adult intracranial volume analysis (1)

**Table S9** Associations of seven early-life head circumference SNPs (*P-*values <5 × 10^-8^) with adult intelligence

| **SNP** | **CHR** | **Position** | **Nearest gene** | **EA/non_EA** | **EAF^a^** | **Beta^a^** | **SE^a^** | **P-value^a^** | **N^a^** |
| --- | --- | --- | --- | --- | --- | --- | --- | --- | --- |
| rs8756 | 12 | 66359752 | *HMGA2* | C/A | 0.45 | 0.001 | 0.003 | 0.002 | 269489 |
| rs9795522 | 12 | 123730935 | *C12orf65* | C/A | 0.23 | 0.02 | 0.003 | 1.41 x 10^-6^ | 265467 |
| rs10883848 | 10 | 104973061 | *NT5C2* | A/G | 0.34 | 0.008 | 0.003 | 0.01 | 262558 |
| rs6095360 | 20 | 47532536 | *ARFGEF2* | G/A | 0.31 | 0.02 | 0.003 | **2.31 x 10^-16^** | 267761 |
| rs3134614 | 1 | 40363054 | *MYCL1* | C/G | 0.88 | -0.001 | 0.004 | 0.90 | 266839 |
| rs6016511 | 20 | 39709255 | *TOP1* | T/C | 0.66 | -0.003 | 0.003 | 0.32 | 269098 |
| rs7792211 | 7 | 50707141 | *GRB10* | G/A | 0.45 | 0.01 | 0.003 | 0.03 | 264700 |

CHR, chromosome; EA, effect allele; EAF, effect allele frequency; SE, standard error.
Bolded *P*-values indicate genome-wide significance.
^a^ From adult intelligence analysis (2)

**Table S10** Associations of seven early-life head circumference SNPs (*P-*values < 5 × 10^-8^) with adult Alzheimer’s disease

| **SNP** | **CHR** | **Position** | **Nearest gene** | **EA/non_EA** | **EAF^a^** | **Beta^a^** | **SE^a^** | **P-value^a^** | **N^a^** |
| --- | --- | --- | --- | --- | --- | --- | --- | --- | --- |
| rs8756 | 12 | 66359752 | *HMGA2* | C/A | 0.46 | -0.01 | 0.002 | 0.02 | 458378 |
| rs9795522 | 12 | 123730935 | *C12orf65* | C/A | - | - | - | - | - |
| rs10883848 | 10 | 104973061 | *NT5C2* | A/G | 0.34 | 0.002 | 0.002 | 0.32 | 451845 |
| rs6095360 | 20 | 47532536 | *ARFGEF2* | G/A | 0.31 | 0.003 | 0.002 | 0.13 | 448548 |
| rs3134614 | 1 | 40363054 | *MYCL1* | C/G | 0.88 | 0.003 | 0.003 | 0.35 | 458744 |
| rs6016511 | 20 | 39709255 | *TOP1* | T/C | 0.66 | 0.0001 | 0.002 | 0.96 | 458532 |
| rs7792211 | 7 | 50707141 | *GRB10* | G/A | 0.45 | 0.001 | 0.002 | 0.01 | 455107 |

CHR, chromosome; EA, effect allele; EAF, effect allele frequency; SE, standard error.
*P*-value< 5 × 10^-8^ is considered statistically significant.
^a^ From adult Alzheimer’s disease analysis (3)

**Table S11** Associations of seven early-life head circumference SNPs (*P-*values <5 × 10^-8^) with adult neuroticism

| **SNP** | **CHR** | **Position** | **Nearest gene** | **EA/non_EA** | **EAF^a^** | **Z-score^a^** | **P-value^a^** | **N^a^** |
| --- | --- | --- | --- | --- | --- | --- | --- | --- |
| rs8756 | 12 | 66359752 | *HMGA2* | C/A | 0.47 | -2.34 | 0.02 | 390278 |
| rs9795522 | 12 | 123730935 | *C12orf65* | C/A | 0.22 | -2.46 | 0.01 | 371787 |
| rs10883848 | 10 | 104973061 | *NT5C2* | A/G | 0.31 | -2.46 | 0.01 | 366260 |
| rs6095360 | 20 | 47532536 | *ARFGEF2* | G/A | 0.33 | 2.51 | 0.01 | 386960 |
| rs3134614 | 1 | 40363054 | *MYCL1* | C/G | 0.88 | -1.25 | 0.21 | 390278 |
| rs6016511 | 20 | 39709255 | *TOP1* | T/C | 0.67 | 0.87 | 0.39 | 390072 |
| rs7792211 | 7 | 50707141 | *GRB10* | G/A | 0.45 | 0.67 | 0.50 | 369421 |

CHR, chromosome; EA, effect allele; EAF, effect allele frequency.
*P*-value< 5 × 10^-8^ is considered statistically significant.
^a^ From adult neuroticism analysis (4)

**Table S12** Associations of seven early-life head circumference SNPs (*P-*values <5 × 10^-8^) with adult depression

| **SNP** | **CHR** | **Position** | **Nearest gene** | **EA/non_EA** | **EAF^a^** | **Z-score^a^** | **P-value^a^** | **N^a^** |
| --- | --- | --- | --- | --- | --- | --- | --- | --- |
| rs8756 | 12 | 66359752 | *HMGA2* | C/A | 0.48 | 1.95 | 0.05 | 381455 |
| rs9795522 | 12 | 123730935 | *C12orf65* | C/A | 0.21 | -1.57 | 0.12 | 361613 |
| rs10883848 | 10 | 104973061 | *NT5C2* | A/G | 0.31 | -1.19 | 0.23 | 356227 |
| rs6095360 | 20 | 47532536 | *ARFGEF2* | G/A | 0.32 | 3.10 | 0.02 | 378220 |
| rs3134614 | 1 | 40363054 | *MYCL1* | C/G | 0.87 | -0.91 | 0.36 | 362696 |
| rs6016511 | 20 | 39709255 | *TOP1* | T/C | 0.68 | 0.99 | 0.32 | 381256 |
| rs7792211 | 7 | 50707141 | *GRB10* | G/A | 0.45 | -1.07 | 0.28 | 359342 |

CHR, chromosome; EA, effect allele; EAF, effect allele frequency.
*P*-value< 5 × 10^-8^ is considered statistically significant.
^a^ From adult depression analysis (4)

**Table S13** Associations of seven early-life head circumference SNPs (*P-*values <5 × 10^-8^) with educational attainment

| **SNP** | **CHR** | **Position** | **Nearest gene** | **EA/non_EA** | **EAF^a^** | **Beta^a^** | **SE^a^** | **P-value^a^** | **N^a^** |
| --- | --- | --- | --- | --- | --- | --- | --- | --- | --- |
| rs8756 | 12 | 66359752 | *HMGA2* | C/A | 0.45 | 0.01 | 0.002 | 1.41 x 10^-6^ | 1131881 |
| rs9795522 | 12 | 123730935 | *C12orf65* | C/A | - | - | - | - | **-** |
| rs10883848 | 10 | 104973061 | *NT5C2* | A/G | 0.30 | 0.01 | 0.002 | 1.65 x 10^-3^ | 1131881 |
| rs6095360 | 20 | 47532536 | *ARFGEF2* | G/A | 0.32 | 0.01 | 0.002 | 1.13 x 10^-5^ | 1131881 |
| rs3134614 | 1 | 40363054 | *MYCL1* | C/G | 0.89 | -0.002 | 0.003 | 0.45 | 1131881 |
| rs6016511 | 20 | 39709255 | *TOP1* | T/C | 0.68 | 0.003 | 0.002 | 0.17 | 1131881 |
| rs7792211 | 7 | 50707141 | *GRB10* | G/A | 0.47 | 0.004 | 0.002 | 0.03 | 1131881 |

CHR, chromosome; EA, effect allele; EAF, effect allele frequency; SE, standard error.
*P*-value< 5 × 10^-8^ is considered statistically significant.
^a^ From adult educational attainment analysis(5)

**Table S14** Associations of seven early-life head circumference SNPs (*P-*values <5 × 10^-8^) with birth head circumference

| **SNP** | **CHR** | **Position** | **Nearest gene** | **EA/non_EA** | **EAF^a^** | **Beta^a^** | **SE^a^** | **P-value ^a^** | **N^a^** |
| --- | --- | --- | --- | --- | --- | --- | --- | --- | --- |
| rs8756 | 12 | 66359752 | *HMGA2* | C/A | 0.50 | 0.027 | 0.008 | 8.33 x 10^-4^ | 28370 |
| rs9795522 | 12 | 123730935 | *C12orf65* | C/A | 0.23 | 0.016 | 0.009 | 0.08 | 32084 |
| rs10883848 | 10 | 104973061 | *NT5C2* | A/G | 0.34 | 0.027 | 0.010 | 8.40 x 10^-3^ | 20591 |
| rs6095360 | 20 | 47532536 | *ARFGEF2* | G/A | 0.33 | 0.014 | 0.008 | 0.10 | 31142 |
| rs3134614 | 1 | 40363054 | *MYCL1* | C/G | 0.88 | 0.022 | 0.014 | 0.12 | 2135 |
| rs6016511 | 20 | 39709255 | *TOP1* | T/C | 0.68 | 0.006 | 0.009 | 0.48 | 30869 |
| rs7792211 | 7 | 50707141 | *GRB10* | G/A | 0.42 | 0.009 | 0.008 | 0.29 | 30740 |

CHR, chromosome; EA, effect allele; EAF, effect allele frequency; SE, standard error.
*P*-value< 5 × 10^-8^ is considered statistically significant.
^a^ From birth head circumference analysis (unpublished data)

**Table S15** Results of the early-life head circumference genetic risk score in the Generation R Study

|  | **Risk score HC** |  |
| --- | --- | --- |
| **Outcomes (SDS)** | **Beta (95% CI)** | **N** |
| Head circumference, first trimester | -0.0003 (-0.03; 0.03) | 1431 |
| Head circumference, second trimester | -0.0004 (-0.03; 0.03) | 1957 |
| Head circumference, third trimester | 0.05 (0.02; 0.08) | 1984 |
| Head circumference, at birth | 0.01 (-0.03; 0.04) | 1366 |
| Head circumference, 1 month | 0.06 (0.03; 0.09) | 1501 |
| Head circumference, 6 months | 0.05 (0.03; 0.08) | 1662 |
| Head circumference, 11 months | 0.05 (0.02; 0.07) | 1528 |
| Head circumference, 6 years | 0.04 (0.02; 0.07) | 4010 |
| Intracranial volume, 64 years | 0.04 (0.03; 0.05) | 22152 |

**Acknowledgements** Cohort specific

**Amsterdam Born Children and their Development**We thank all participating hospitals, obstetric clinics, general practitioners and primary schools for their assistance in implementing the ABCD study. We also gratefully acknowledge all the women and children who participated in this study for their cooperation. **Avon Longitudinal Study of Parents and Children (ALSPAC)**We are extremely grateful to all the families who took part in this study, the midwives for their help in recruiting them, and the whole ALSPAC team, which includes interviewers, computer and laboratory technicians, clerical workers, research scientists, volunteers, managers, receptionists and nurses. GWAS data was generated by Sample Logistics and Genotyping Facilities at Wellcome Sanger Institute and LabCorp (Laboratory Corporation of America) using support from 23andMe. The UK Medical Research Council and Wellcome (Grant ref: 102215/2/13/2) and the University of Bristol provide core support for ALSPAC. This publication is the work of the authors who will serve as guarantors for the contents of this paper. A comprehensive list of grants funding is available on the ALSPAC website at (<http://www.bristol.ac.uk/alspac/external/documents/grant-acknowledgements.pdf>).

**Children’s Hospital of Philadelphia (CHOP)**The authors thank the network of primary care clinicians and the patients and families for their contribution to this project and to clinical research facilitated by the Pediatric Research Consortium [PeRC]-The Children’s Hospital of Philadelphia. R. Chiavacci, E. Dabaghyan, A. [Hope] Thomas, K. Harden, A. Hill, C. Johnson-Honesty, C. Drummond, S. Harrison, F. Salley, C. Gibbons, K. Lilliston, C. Kim, E. Frackelton, F. Mentch, G. Otieno, K. Thomas, C. Hou, K. Thomas and M.L. Garris provided expert assistance with genotyping and/or data collection and management. The authors would also like to thank S. Kristinsson, L.A. Hermannsson and A. Krisbjörnsson of Raförninn ehf for extensive software design and contributions. This research was financially supported by an Institute Development Award from the Children’s Hospital of Philadelphia, a Research Development Award from the Cotswold Foundation, the Daniel B. Burke Endowed Chair for Diabetes Research, the Children’s Hospital of Philadelphia Endowed Chair in Genomic Research and NIH grant R01 HD056465.

**CHOP Europe**The authors thank the participating families and all project partners for their enthusiastic support of the project work. We thank Dr Eva Reischl and team at the Genome Analysis Center of Helmholtz Zentrum Muenchen for gentopyping and intial QC of the data and Dr Linda Broer and team at the Department of Internal Medicine, Genetic Laboratory, Erasmus Medical Center, Rotterdam, The Netherlands for extensive QC and imputation of the genotyped data. We also like to acknowledge the The European Childhood Obesity Trial Study Group for their continuous and salient support of the CHOP project: Philippe Goyens, Clotilde Carlier, Joana Hoyos, Pascale Poncelet, and Elena Dain (Universite Libre de Bruxelles – (ULB) –Brussels , Belgium); Jean-Noel Van Hees (CHC St Vincent– Françoise Martin, Annick Xhonneux, Jean-Paul Langhendries, and Jean-Noel Van Hees - Liège-Rocourt, Belgium); Ricardo Closa-Monasterolo, Joaquin Escribano, Veronica Luque, Georgina Mendez, Natalia Ferre, and Marta Zaragoza-Jordana (Universitat Rovira i Virgili, Institut d’Investigacio´ Sanitaria Pere Virgili, Taragona, Spain); Marcello Giovannini, Enrica Riva, Carlo Agostoni, Silvia Scaglioni, Elvira Verduci, Fiammetta Vecchi, and Alice Re Dionigi (University of Milano, Milano, Italy); Jerzy Socha, Piotr Socha and Anna Stolarczyk (Children’s Memorial Health Institute, Department of Gastroenterology, Hepatology and Immunology, Warsaw, Poland); Anna Dobrzanska and Dariusz Gruszfeld (Children’s Memorial Health Institute, Neonatal Intensive Care Unit, Warsaw, Poland); Roman Janas (Children’s Memorial Health Institute, Diagnostic Laboratory, Warsaw, Poland); Emmanuel Perrin (Danone Research Centre for Specialized Nutrition, Schiphol, the Netherlands); Rudiger von Kries (Division of Pediatric Epidemiology, Institute of Social Pediatrics and Adolescent Medicine, Ludwig Maximilians University of Munich, Munich, Germany); Helfried Groebe, Anna Reith, and Renate Hofmann (Klinikum Nurnberg Sued, Nurnberg, Germany); and Berthold Koletzko, Veit Grote, Martina Weber, Peter Rzehak, Sonia Schiess, Jeannette Beyer, Michaela Fritsch, Uschi Handel, Ingrid Pawellek, Sabine Verwied-Jorky, Iris Hannibal, Hans Demmelmair, Gudrun Haile, and Melissa Theurich (Division of Nutritional Medicine and Metabolism, Dr von Hauner Childrens Hospital, Ludwig-Maximilians Universität München (LMU), Munich, Germany).

**Copenhagen Prospective Studies on Asthma in Childhood- COPSAC 2000 Cohort**We express our deepest gratitude to the children and families of the COPSAC 2000 cohort study for all their support and commitment. We acknowledge and appreciate the unique efforts of the COPSAC research team.

**Copenhagen Prospective Studies on Asthma in Childhood- COPSAC 2010 Cohort**We express our deepest gratitude to the children and families of the COPSAC 2010 cohort study for all their support and commitment. We acknowledge and appreciate the unique efforts of the COPSAC research team.

**Copenhagen Prospective Studies on Asthma in Childhood- COPSAC Registry**We express our deepest gratitude to the children and families of the COPSAC Registry cohort study for all their support and commitment. We acknowledge and appreciate the unique efforts of the COPSAC research team.

**DNBC GOYA-offspring**We are thankful for all DNBC mothers and children that have contributed with their time and effort in order to establish this unique data source for research.

**The Danish National Birth Cohort-Preterm Birth Study (DNBC-PTB)**
We are very grateful to all DNBC families who took part in the study. We would also like to thank everyone involved in data collection and biological material handling.

**EFSOCH**We are grateful to all EFSOCH participants and to the EFSOCH study team. The authors would like to acknowledge the use of the University of Exeter High-Performance Computing (HPC) facility in carrying out this work**.

Generation R**The Generation R Study is conducted by the Erasmus Medical Center in close collaboration with the School of Law and Faculty of Social Sciences of the Erasmus University Rotterdam, the Municipal Health Service Rotterdam area, Rotterdam, the Rotterdam Homecare Foundation, Rotterdam and the Stichting Trombosedienst & Artsenlaboratorium Rijnmond (STAR-MDC), Rotterdam. We gratefully acknowledge the contribution of children and parents, general practitioners, hospitals, midwives and pharmacies in Rotterdam. The study protocol was approved by the Medical Ethical Committee of the Erasmus Medical Centre, Rotterdam. Written informed consent was obtained from all participants. The generation and management of GWAS genotype data for the Generation R Study were done at the Genetic Laboratory of the Department of Internal Medicine, Erasmus MC, The Netherlands. We would like to thank Karol Estrada, Dr. Tobias A. Knoch, Anis Abuseiris, Luc V. de Zeeuw, and Rob de Graaf, for their help in creating GRIMP, BigGRID, MediGRID, and Services@MediGRID/D-Grid, (funded by the German Bundesministerium fuer Forschung und Technology; grants 01 AK 803 A-H, 01 IG 07015 G) for access to their grid computing resources. We thank Mila Jhamai, Manoushka Ganesh, Pascal Arp, Marijn Verkerk, Lizbeth Herrera and Marjolein Peters for their help in creating, managing and QC of the GWAS database. Also, we thank Karol Estrada for their support in creation and analysis of imputed data.

**HAPO**We would like to acknowledge the participants and research personnel at the participating HAPO field centres.

**HBCS**
We thank all study participants as well as everybody involved in the Helsinki Birth Cohort Study.
 **INfancia y Medio Ambiente [Environment and Childhood] (INMA) Project**The authors would like to thank all the participants for their generous collaboration. A full roster of the INMA Project Investigators can be found at <http://www.proyectoinma.org/presentacion-inma/listado-investigadores/en_listado-investigadores.html>.

**IOW**
The author would like to thank all the participants for their valuable effort in maintaining the cohort. We would like to acknowledge the cooperation of the ISLE of Wight 1989 birth cohort participant and their families who have helped us with this ongoing project for the past two decades.

**LIFE-Child**We thank all children and families who participated in the studies. We gratefully appreciate the help of the study nurses, technical assistants and physicians who performed the clinical examinations and data collection.
 **Lifestyle – Immune System – Allergy Study and German Infant Study on the influence of Nutrition Intervention (LISA+GINI)**The authors thank all the families for their participation in the GINIplus and LISA studies. Furthermore, we thank all members of the GINIplus and LISA Study Groups for their excellent work. The GINIplus Study group consists of the following: Institute of Epidemiology, Helmholtz Zentrum München, German Research Center for Environmental Health, Neuherberg (Heinrich J, Brüske I, Schulz H, Flexeder C, Zeller C, Standl M, Schnappinger M, Ferland M, Thiering E, Tiesler C); Department of Pediatrics, Marien-Hospital, Wesel (Berdel D, von Berg A); Ludwig-Maximilians-University of Munich, Dr von Hauner Children’s Hospital (Koletzko S); Child and Adolescent Medicine, University Hospital rechts der Isar of the Technical University Munich (Bauer CP, Hoffmann U); IUF- Environmental Health Research Institute, Düsseldorf (Schikowski T, Link E, Klümper C, Krämer U, Sugiri D). The LISA Study group consists of the following: Helmholtz Zentrum München, German Research Center for Environmental Health, Institute of Epidemiology, Munich (Heinrich J, Schnappinger M, Brüske I, Ferland M, Schulz H, Zeller C, Standl M, Thiering E, Tiesler C, Flexeder C); Department of Pediatrics, Municipal Hospital “St. Georg”, Leipzig (Borte M, Diez U, Dorn C, Braun E); Marien Hospital Wesel, Department of Pediatrics, Wesel (von Berg A, Berdel D, Stiers G, Maas B); Pediatric Practice, Bad Honnef (Schaaf B); Helmholtz Centre of Environmental Research – UFZ, Department of Environmental Immunology/Core Facility Studies, Leipzig (Lehmann I, Bauer M, Röder S, Schilde M, Nowak M, Herberth G , Müller J); Technical University Munich, Department of Pediatrics, Munich (Hoffmann U, Paschke M, Marra S); Clinical Research Group Molecular Dermatology, Department of Dermatology and Allergy, Technische Universität München (TUM), Munich (Ollert M, J. Grosch).  **Northern Finnish Birth Cohort Study 1966 (NFBC66) and 1968 (NFBC86)**We thank Professor Paula Rantakallio (launch of NFBC1966 and 1986), Ms Outi Tornwall and Ms Minttu Jussila (DNA biobanking). Financial support was received from the Academy of Finland (project grants 104781, 120315, 129269, 1114194, Center of Excellence in Complex Disease Genetics and SALVE), University Hospital Oulu, Biocenter, University of Oulu, Finland (75617), the European Commission (EURO-BLCS, Framework 5 award QLG1-CT-2000-01643), NHLBI grant 5R01HL087679-02 through the STAMPEED program (1RL1MH083268-01), NIH/NIMH (5R01MH63706:02), the Medical Research Council, UK (G0500539, G0600705, PrevMetSyn/SALVE) and the Wellcome Trust (project grant GR069224), UK., ENGAGE project and grant agreement HEALTH-F4-2007- 201413. The DNA extractions, sample quality controls, biobank up-keeping and aliquotting was performed in the National Public Health Institute, Biomedicum Helsinki, Finland and supported financially by the Academy of Finland and Biocentrum Helsinki.

**Norwegian Mother and Child (MoBa) Cohort Study  (Pål R. Njølstad Bo Jacobsson)**The Norwegian Mother and Child Cohort Study are supported by the Norwegian Ministry of Health and Care Services and the Ministry of Education and Research, NIH/NIEHS (contract no N01-ES-75558), NIH/NINDS (grant no.1 UO1 NS 047537-01 and grant no.2 UO1 NS 047537-06A1). We are grateful to all the participating families in Norway who take part in this on-going cohort study.
 **The Physical Activity and Nutrition in Children (PANIC) Study**We are grateful for children, adolescents, and their families who have participated in The PANIC Study since the pilot study in 2006. We also thank all researchers and auxiliary personnel who have in many ways carried out The PANIC Study.
 **Project Viva**Project Viva investigators and research team thank the mothers and children that have been participating to Project Viva throughout the years.

**The Raine Study**This study was supported by the National Health and Medical Research Council of Australia [grant numbers 572613, 403981 and 003209] and the Canadian Institutes of Health Research [grant number MOP-82893]. The authors are grateful to the Raine Study participants and their families, and to the Raine Study research staff for cohort coordination and data collection. The authors gratefully acknowledge the NH&MRC for their long term contribution to funding the study over the last 30 years and also the following Institutions for providing funding for Core Management of the Raine Study: The University of Western Australia (UWA), Curtin University, Raine Medical Research Foundation, Telethon Kids Institute, and Women and Infants Research Foundation, Murdoch University, The University of Notre Dame (Australia), and Edith Cowan University. The authors gratefully acknowledge the assistance of the Western Australian DNA Bank (National Health and Medical Research Council of Australia National Enabling Facility).

This work was supported by resources provided by the Pawsey Supercomputing Centre with funding from the Australian Government and the Government of Western Australia. This publication is the work of authors, and hence serve as guarantors for the information of this study in this paper.

**SKOT (1 & 2)**The authors wish to thank all children and parents that were part of the SKOT 1 and 2 study.

**Special Turku Coronary Risk Factor Intervention Project (STRIP)**We thank all the study participants and their families. The study was approved by the Joint Commission on Ethics of the Turku University and the Turku University Central Hospital. Informed consent was obtained from all parents at the beginning of the trial and from the children at 15 and 18 years of age.

**TEENS of Attica: Genes and Environment Study (TEENAGE)**We would like to thank all study participants and their families as well as all volunteers for their contribution in this study.  We thank the Sample Management and Genotyping Facilities staff at the Wellcome Trust Sanger Institute for sample preparation, quality control and genotyping.

**TDCOB**The Danish Childhood Obesity Biobank would like to thank all children, youths, and their families for participation in the studies and thus providing opportunities to detect novel insights in regards to childhood obesity. We wish to think Mrs. Oda Troest and Mrs. Birgitte Holløse for their invaluable assistance with blood samples and database. This study is part of the research activities in TARGET (The Impact of our Genomes on Individual Treatment Response in Obese Children, www.target.ku.dk), and BIOCHILD (Genetics and Systems Biology of Childhood Obesity in India and Denmark, www.biochild.ku.dk). The study is part of The Danish Childhood Obesity Biobank; ClinicalTrials.gov ID-no.: NCT00928473. **UK Biobank**This research has been conducted using the UK Biobank Resource under Application Number 23509.

**Methods** Cohort information

**Amsterdam Born Children and their Development***Study design*

The Amsterdam Born Children and their Development (ABCD) cohort study is a large, community-based birth cohort, which started in 2003 with the inclusion of 8,000 pregnant women living in Amsterdam and its main aim is to study factors in early life (during pregnancy and infancy) that explain health later in life. Detailed information on life style habits, psychosocial determinants, obstetric complications, blood pressure course during pregnancy, as well as childhood growth patterns has been gathered (6). Data for this study comes from ABCD-Genetic Enrichment (ABCD-GE) study, a sub-study of 1192 ethnic Dutch children. The blood was collected from a simple finger prick during the 5-year health check-up of the children (2008-2010). DNA was extracted from the dried blood spots (7). Approval of the study was obtained from the Central Committee on Research Involving Human Subjects in The Netherlands, the medical ethics review committees of the participating hospitals and the Registration Committee of the Municipality of Amsterdam. Approval of the ABCD-GE study was obtained from the Medical Ethical Committees of the Academic Medical Centre in Amsterdam. For the ABCD-GE study, an opt out procedure was followed. Written consent was obtained from participating parents.

DNA samples were genotyped using the Illumina Human Core Exom Beadchip (Illumina, San Diego, CA, USA), which includes over 540,000 genetic markers. Before imputation, SNPs were excluded if they had high levels of missing data (SNP call rate < 95%), strong departures from Hardy-Weinberg equilibrium (P<1 x 10^-^*6*), or low Minor allele frequencies (MAF) (<1%). Individuals were excluded if mismatch in heterozygosity, gender or relatedness existed. Genetic markers were imputed using the IMPUTE2-software and the 1000 Genomes References Panel (phase 1 release v3, build 37). Genotypes for the SNPs of interest were extracted from the imputed Genome wide association study (GWAS)-dataset. The final number of SNPs was 277,644. The total of SNPs after imputation was 27,448,454.

Genome-wide analyses were performed using SNPTEST v.2.5 (mathgen.stats.ox.ac.uk/genetics_software/snptest/snptest.html) using dosages of alternate allele with an additive linear model using four principal components as covariates (HC_INFANT SDS ~ SNP + 4 principal components).

**Avon Longitudinal Study of Parents and Children (ALSPAC)**ALSPAC is a prospective birth cohort which recruited pregnant women with expected delivery dates between April 1991 and December 1992 from Bristol UK. 14,541 pregnant women were initially enrolled with 14,062 children born. Detailed information on health and development of children and their parents were collected from regular clinic visits and completion of questionnaires. A detailed description of the cohort has been published previously (8, 9). The study website contains details of all the data that is available through a fully searchable data dictionary: <http://www.bris.ac.uk/alspac/researchers/data-access/data-dictionary/>. Ethical approval was obtained from the ALSPAC Law and Ethics Committee and the Local Ethics Committees.

**ALSPAC GWAS data**A total of 9,912 children were genotyped using the Illumina HumanHap550 quad genome-wide SNP genotyping platform by the Wellcome Trust Sanger Institute, Cambridge, UK and the Laboratory Corporation of America, Burlington, NC, USA using support from 23andMe. Details on the QC procedure have been published previously (10). In short, after quality control (individual call rate>0.97, SNP call rate>0.95, minor allele frequency>0.01, Hardy-Weinberg equilibrium *p*>5x10^-7^, and removal of individuals with cryptic relatedness and non-European ancestry), 8,237 children and 477,482 SNP genotypes were retained. SNPs were flipped to the forward strand and haplotypes were estimated using ShapeIT (v2.r644). Imputation was performed using Impute V2.2.22 to a 1000 genomes reference haplotype template (Version 1 Phase 3.

**Children’s Hospital of Philadelphia (CHOP)**The CHOP study was genotyped at the Center for Applied Genomics at the Children’s Hospital of Philadelphia. The Research Ethics Board of CHOP approved the study, and written informed consent was obtained from all subjects. The children were genotyped on the Illumina HumanHap550 and 610 quad SNP genotyping platforms. Plink was used for the initial quality control where SNPs were excluded with minor allele frequency < 0.01, Hardy-Weinberg equilibrium p-value < 1.0x10^-6^, and genotyping call rate < 0.05. Children were removed for non-European American ancestry and individual call rate < 0.05. ShapeIT v2 was used to pre-phase each chromosome prior to imputation with impute2. The 1000 genomes project version 1 phase 3 was used as a reference panel for the imputation. Snptest was used for to assess statistical significance with the phenotype. **CHOP Europe**The CHOP study (European Childhood Obesity Project) is an ongoing European multicenter randomized prospective nutritional intervention study in 1678 healthy term newborns recruited between October 1, 2002 and July 31, 2004. Currently, infants are followed up until the age of 11 years. Main objective of the CHOP study is to assess the effect of early and later nutrition on children’s weight development, growth, body composition and risk of obesity and the role of genetic variation and epigenetic and metabolic programming plays in this context. A detailed description of the study design and the comprehensive prospective measurements can be found in recent publications (11-15). The local ethics committees of each study center approved all study procedures: Belgium (Comitè d’Ethique de L’Hopital Universitaire des Enfants Reine Fabiola; no. CEH 14/02), Germany (Bayerische Landesärztekammer Ethik-Kommission; no. 02070), Italy (Azienda Ospedaliera San Paolo Comitato Etico; no. 14/2002), Poland (Instytut Pomnik–Centrum Zdrowia Dziecka Komitet Etyczny; no 243/KE/2001), and Spain (Comité ético de investigación clinica del Hospital Universitario de Tarragona Joan XXIII). Written informed parental consent was obtained for each participating infant and from children of age 8 years onwards.

***Genotyping , Quality Control and Imputation***

For this genome-wide-association analysis (GWAS) genetic data on n=374 children were available from the CHOP study. Buffy coats were collected from children of age 5.5 years during physical exam. Samples were genotyped from genomic DNA extracted from buffy coats with Illumina HumanOmniExpress-24 v1.0 arrays by Dr Eva Reischl and team at the Genome Analysis Center of Helmholtz Zentrum Muenchen, Germany by standard procedures according to manufactures instructions (Illumina Inc., San Diego, USA).
 Quality control on genotyped data were performed at Helmholtz Zentrum Muenchen and in even more detail by Dr Linda Broer and team at the Department of Internal Medicine, Genetic Laboratory, Erasmus Medical Center, Rotterdam, The Netherlands. QC tests using mainly PLINK comprised SNP missingness test (GENO>0.05), frequency test (MAF<0.001), HWE-test (p<=1 × 10-7), Sample call rate >95% and SNP call rate >80% and Sample call rate >97.5% and SNP call rate >95%, test for evidence of heterozygosity or excess homozygosity, test for gender mismatch, test for Caucasian samples and test for familiar relationships (IBD). From the originally n=382 samples with 701,281 SNPs 264 SNPS failed the HWE-test (p<=1 × 10-7) and 14,627 SNPs failed the MAF test (<0.1%) leaving 686,390 SNPs. For 8 samples there was evidence of excess heterogeneity (F-value < mean –(4×SD)) and these samples were removed leaving n=374 samples with 686,390 SNPs. No gender mismatches were identified. However, 10 of the 374 samples were identified as potentially non-European by IBS/IBD distance analysis in PLINK but kept in the data set. This issue was accounted for by including 10 pruned principal components from multidimensional scaling procedure in the GWAS analysis as adjustment factors (see below). Overall , quality controlled genotyped data from 374 children with 686,390 SNPs were available.

Imputation of these quality controlled genotyped data with reference to the 1000G reference panel (phase1_v3) were performed by Dr Linda Broer and team at the Department of Internal Medicine, Genetic Laboratory, Erasmus Medical Center, Rotterdam, The Netherlands. Imputation was done using a two steps procedure using MINIMAC3 and MACH for phasing and imputation respectively for the 374 children and by using the Michigan Imputation server (https://imputationserver.sph.umich.edu/index.html). The final number of SNPs with MAF>1% and r2 >0.3 after the imputation process is 9,642,852. MACH R² when excluding variants MAF < 1% is 0.88 (median = 0.97, SD = 0.20).

***Conducted analysis and description of covariates***

Statistical analysis is based on all genotyped and imputed data (9,642,852 SNPs over 22 chromosomes) for n=374 children. However due to missing data in phenotype (standardized head circumference at birth, respectively at infancy) analysis is based on n=361, respectively n=366 children only.

**Copenhagen Prospective Studies on Asthma in Childhood (COPSAC) Cohorts (COPSAC 2000, COPSAC 2010 and COPSAC registry):**

**COPSAC 2000 Cohort (16)**

The COPSAC 2000 cohort is a prospective clinical mother-child cohort study of 411 children of asthmatic mothers. The study inclusion criteria included mothers fluent in Danish and a physician’s diagnosis of asthma and the need of daily treatment of asthma after the age of 7 years. Exclusion criteria were failure to show up at this first visit, severe congenital anomaly, gestational age <36 weeks, and mechanical ventilation or lower respiratory tract infection prior to enrollment. 411 infants were enrolled in the cohort at one month of age between Aug 1998 and Dec 2001. The study was conducted in accordance with the Declaration of Helsinki and was approved by the Copenhagen Ethics Committee and the Danish Data Protection Agency.

**COPSAC 2010 Cohort (17)**

The COPSAC 2010 mother-child cohort is a population based longitudinal clinical study of 736 pregnant women and their 700 children. Recruitment of 736 pregnant women was initiated late 2008 and ended July 2010 with 700 children enrolled after the last birth in April 2011. The study was conducted in accordance with the Declaration of Helsinki and was approved by the Copenhagen Ethics Committee and the Danish Data Protection Agency. 

**COPSAC Registry Cohort (18, 19)**

This is a register based cohort comprising children with asthma who have been characterized from the national health registries. In accordance with the Danish law, the research ethics committee can grant exemption from obtaining informed consent under certain circumstances. For this study cohort, such an exemption was granted (H-B-2998-103).

The study was conducted in accordance with the Declaration of Helsinki and was approved by the Copenhagen Ethics Committee and the Danish Data Protection Agency.

**Head circumference measures:** Head circumference was measured with a tape, using the largest diameter as measurement.

**Genotyping (19):** Genotyping of 951,117 genetic markers was performed using the Illumina Infinium HumanOmniExpressExome Bead chip at the AROS Applied Biotechnology AS center, in Aarhus, Denmark. Genotypes were called with Illumina's Genome Studio software. All 2,339 individuals (COPSAC2000, COPSAC2010, COPSAC Registry cohorts) underwent quality control (QC) filters, where individuals with Hardy-Weinberg equilibria p values >10^-6^, minor allele frequency >0.01, individual genotyping call rate >0.95, and SNP genotyping call rate >0.95 were retained. We excluded individuals with gender mismatches, genetic duplicates, outlying heterozygosity >0.27 and <0.037, and those not clustering with the HapMap CEU individuals (Utah residents with ancestry from northern and Western Europe) through a multi-dimensional clustering analyses (MDS) seeded with individuals from the International Hap Map Phase 3. After QC filtering, 2,209 individuals with 657,699 markers remained. Imputation to 1000 Genomes reference panel using SHAPEIT2 and IMPUTE2 was carried out at the Barcelona Supercomputing Center (www.bsc.es). 

**DNBC GOYA-offspring**The Danish National Birth cohort (DNBC) is a population-based prospective birth cohort study that was established in 1996-2002 and enrolled a total of 100,417 pregnancies among 92,274 women from all over Denmark (20). In the present analysis, children of two selected groups were included: children from randomly selected mothers of the cohort and children born to mothers with obesity. Head circumference at birth was measured by midwives and obtained from the Danish National Birth Registry. Measurement of head circumference at 12 months was conducted by the general practitioner or a health nurse and was written in the ‘child’s book’, a health record used in Denmark to communicate between health visitors, the parents and the general practitioner. The book was kept with the parents and the information was then reported by the mother during the telephone interview 18 months postpartum.

    Cord blood was collected at birth. The children were genotyped using the Illumina Infinium HumanCoreExome Beadchip (Illumina, San Diego, CA, USA) and genotypes were called using the Genotyping module, version 1.9.4 of GenomeStudio software, version 2011.1 (Illumina, San Diego, CA, USA). During quality control, we excluded closely related individuals and samples with extreme inbreeding coefficients, mislabeled gender, or call rate <95% as well as duplicates and individuals identified as ethnic outliers. We applied a >95% genotype call rate filer for the inclusion of SNPs. Additional genotypes were imputed into 1000 Genomes Phase 1 using Impute 2. 

**The Danish National Birth Cohort-Preterm Birth Study (DNBC-PTB)** is a case-control study using mother-infant pairs to investigate genetic and environmental influences on spontaneous preterm birth. The study is nested within the Danish National Birth Cohort (DNBC), a population-based cohort of more than 100,000 pregnancies, recruited in the years 1996-2002 (20). Extensive phenotype information was collected by computer-assisted telephone interviews twice during pregnancy as well as 6 and 18 months after delivery. Additional questionnaire-based follow-up surveys are conducted at regular intervals, and further information on traits and diseases can be obtained through linkage with Danish health and population registers. The DNBC mothers provided written informed consent on behalf of themselves and their children. The study protocol was approved by the Regional Scientific Ethical Committee of Copenhagen and the Danish Data Protection Agency.

Head circumference at birth measured by midwives was retrieved from the Danish Medical Birth Register. Infant head circumference was measured at the child’s routine visit to the general practitioner around 12 months of age, and the information was reported by the mother during DNBC telephone interview 4, conducted approximately 18 months after birth.

GWAS data were obtained using the Illumina Human 660W Quad array and generated as part of the Gene Environment Association Studies (GENEVA) consortium. Data cleaning and QC steps were based on the following requirements for samples and SNPs to be included: sample missingness rate < 4%, heterozygosity with 3 SDs from the mean, variant missingness rate < 2%, MAF > 1%, HWE *P* value > 1 × 10^-6^, A/T and C/G variants excluded. We used a two-step procedure to impute unobserved genotypes using phased haplotypes from the integrated Phase I release of the 1000 Genomes Project (21) (v3.20101123, ALL populations, no monomorphic/singletons). In a first prephasing step, we used SHAPEIT (22) to estimate the haplotypes for our study samples. In a second step, we imputed the missing alleles for additional SNPs directly onto these phased haplotypes using IMPUTE2 (23). Genome-wide association analysis was carried out using SNPTEST v.2.4.1(24) under an additive genetic model.

**EFSOCH**The Exeter Family Study of Childhood Health (EFSOCH) (25) is a prospective study of children born between 2000 and 2004, and their parents, from a geographically defined region of Exeter, UK. All women of self-reported white European ancestry and their partners, living in central Exeter, with a singleton, non-diabetic pregnancy were eligible for enrolment. All women gave informed consent, and ethical approval was obtained from the local review committee.
 The head circumference measures analysed in the current study were obtained at birth and at 1 year of age. At birth, head circumference was measured as soon as possible after delivery to the nearest 0.1 cm using a short fibreglass tape (range up to 61 cm x 0.1 cm). Head circumference at 1 year was measured in the same way. All measures were repeated three times and an average value calculated for use in analyses.
 Maternal and paternal DNA samples were extracted from parental blood samples obtained at the study visit (when the women were 28 weeks pregnant), and offspring DNA was obtained from cord blood at birth.
 Genotyping of 2768 EFSOCH samples (n=969 mothers, 937 fathers and 862 children) was performed using the Illumina Infinium HumanCoreExome-24 array (n=551,839 SNPs/indels). Individuals with genotype call rate <98% were removed (n=50 individuals [1.8%]). SNPs were removed if they had call rates <95% (n=13,151 SNPs), showed evidence of deviation from Hardy-Weinberg equilibrium (P<1x10^-6^; n=455 further SNPs), or had a minor allele frequency (MAF) <1% (n=257,289 further SNPs). Genotypically-derived sex information was compared with sex information in the phenotype file and mismatched samples were excluded (n=13 individuals [0.61%]). Kinship was estimated using King (26). Where evidence of labelling errors was clear, labels were updated, otherwise samples with kinship errors were excluded (n=22 individuals [0.79%]). Principal component analysis was performed to assess ancestry of the sample using flashPCA (27). Outliers were defined as >4.56 SD from the cluster mean (defined using 1000 Genomes European data as the reference) and excluded (n=21 individuals [0.76%]). Genotype data was phased using SHAPEIT2, using the duohmm flag to account for known family relationships. Imputation was carried out using IMPUTE2 and samples were imputed to the 1000Genomes Phase 3 reference panel.
 SNPs were included in the analysis if imputation quality score was >0.4 and MAF ≥1%. In the current study, EFSOCH contributed replication data: A total of 39 SNPs from the imputed dataset were analysed in a total of 584 children with head circumference at 1 year. Analyses were adjusted for genotyping batch.

**Generation R**The Generation R Study is a population-based prospective cohort study from fetal life until young adulthood. All children were born between April 2002 and January 2006. This study is designed to identify early environmental and genetic determinants of growth, development and health from fetal life until young adulthood and has been described previously in detail (28). Detailed measurements were performed using ultrasound and physical examinations, biological samples and advanced imaging techniques. The study has been approved by the Medical Ethics Committee of the Erasmus Medical Center, Rotterdam. Written informed consent was obtained from all participants.
 Cord blood for DNA isolation was available in 58% of all live-born participating children. Sex-mismatch rate between genome based sex and midwife-record based sex was low (<0.5%), indicating that possible contamination of maternal DNA was extremely low. Missing cord blood samples were mainly due to logistical constraints at the delivery. Genome-wide association scans (GWAs) were run using the Illumina 610 Quad and 660 platforms (20). MACH (version 1.0.15) software wa s used to impute genotypes to the 1000 Genomes (March 2010 release) cosmopolitan panel (29, 30). Before imputation, SNPs were excluded if they had high levels of missing data (SNP call rate 98%), strong departures from Hardy-Weinberg equilibrium (*P*-value 1 x10^-6^), or low MAF (1%) (28).

**HBCS**The Helsinki Birth Cohort Study (HBCS) is a population-based prospective cohort of singletons born at the Helsinki University Central Hospital between the years 1934 and 1944. Birth records on 4630 men and 4130 women who lived in Finland in 1971 were taken, including measurements of weight and length. Serial measurements of height and weight were extracted from child welfare clinic and school health clinic records, with an average of ten measurements between birth and 2 years, and eight measurements between 2 and 11 years of age. Between 2000 and 2002, a representative subset of 928 males and 1075 females returned for clinical examinations. At this visit, blood was taken for DNA extraction. Genotyping was performed on a custom Illumina 670 Quad platform at the Wellcome Trust Sanger Centre. Quality control was performed before imputation (excluding genotypes with call rate <0.95, MAF <0.01, HWE *P*-value <1 x 10^-6^) with MACH. The current analysis includes 1551 males and females (43% male) with BMI adjusted to their exact birthday at age 10. Informed consent was collected from all study participants. The study design was approved by the local ethics committee.

**INfancia y Medio Ambiente [Environment and Childhood] (INMA) Project**

Population-based birth cohorts were established as part of the INMA – INfancia y Medio Ambiente [Environment and Childhood] Project in several regions of Spain following a common protocol. This project aims to study the associations between pre- and postnatal environmental exposures and growth, health, and development from early foetal life until adolescence and has been described previously in detail (31). Pregnant women were enrolled during the 1st trimester of pregnancy at public primary health care centers or public hospitals. Detailed measurements were performed using ultrasound and physical examinations and biological samples were collected. Informed consent was obtained from all participants and the study was approved by the Hospital Ethics Committees in each participating region.

This particular analysis uses the INMA cohorts of Menorca (MEN), Valencia (VAL), and Sabadell (SAB) in the discovery phase, and of Gipuzkoa (GIP) in the replication phase. Analyses were restricted to individuals of European ethnic origin with genome-wide data and a head circumference measurement at birth (N=942 discovery phase; N=383 replication phase)  or during infancy (N=550 discovery phase; N=319 replication phase). Head circumference at birth was assessed by a nurse when the newborn arrived at the hospital ward within the first 12 h of life. In all cohorts, except for INMA MEN, head circumference measurements at 1–1.5 years of age were carried out by trained fieldwork nurses. All head circumference measurements were maximum fronto-occipital circumference using a flexible tape following a standardized protocol with no inter-observer reliability measurements required due to a low degree of task complexity (32). In INMA MEN, head circumference measurements at infancy were obtained from medical records.

DNA was obtained from cord blood, whole blood collected at 4y or saliva using the Chemagen protocol at the Spanish National Genotyping Centre (CEGEN). Children whose parents reported to be white and to be born in Spain or in European countries and that were not lost during the follow-up were selected for genotyping. Genome-wide genotyping was performed using the HumanOmni1-Quad Beadchip (Illumina) at CEGEN (MEN, SAB, VAL cohorts) and GSA Beadchip (Illumina) at the Human Genotyping Facility (HuGeF), Dept Internal Medicine, Erasmus MC, The Netherlands (GIP subcohort). Genotype calling was done using the GeneTrain2.0 algorithm based on HapMap clusters implemented in the GenomeStudio software. Quality control was done using PLINK and following standard criteria. First of all, SNPs were flipped to the human genome + strand. We applied the following initial quality control thresholds: sample call rate>98% and/or LRR SD<0.3. Then, we checked sex, relatedness, heterozygosity and population stratification. Genetic variants were filtered for SNP call rate>95%, MAF>1% and HWE *P*-value> 1.10E-06. Imputation of genetic variants was done using IMPUTE V2 and the cosmopolitan 1000 genome panel (release March 2012) (MEN, SAB, VAL) and the Michigan imputation server using the Haplotype Reference Consortium HRC v1.1 reference panel (GIP).

**Isle of Wight (IOW)**

*Cohort description and genotyping*Isle of Wight (IOW) birth cohort is a multigenerational study that was established on the Isle of Wight, UK in January 1989. Second generation children born between 2010-2014 with available information on birth head circumference and genotype were used for this replication study. These children were followed at 6 and 12 months of age. Birth head circumference was measured right after birth. Participants with <37 weeks of gestational age and missing information on the head circumference and genotype were excluded from the analysis. Outliers based on their standard deviation were excluded.

Cordblood samples of the participants were collected at birth. Human OminoExpressExome-8-v1-2-B platform was used for genotyping. Samples with call rate <95% were excluded.  Imputation was carried out using IMPUTE V2 software, using the 1000G reference (phase 1, v3).

*Statistical analysis*All analyses were performed in PLINK and SAS 9.3. We applied linear regression models to test the association between the birth head circumference and SNPs that are identified in the IOW cohort.

**LIFE-Child**LIFE Child (NCT02550236) is an ongoing regional population-based study with comprehensive phenotyping including anthropometric and clinical data, laboratory data, genetic data and psychoscocial assessment in children and parents conducted in the city of Leipzig, Germany (33, 34). As a part of LIFE, the Leipzig Research Center for Civilization Diseases, it aims to monitor healthy child development from birth to adulthood and to understand the development of civilization diseases such as obesity. LEIPZIG childhood obesity cohort/LIFE Child Obesity is an obesity enriched subset with additional metabolic and cardiovascular phenotyping including OGTT. All procedures are performed in accordance with the ethical standards of the institutional and/or national research committee and with the 1964 Helsinki declaration and its later amendments or comparable ethical standards. All legal guardians gave written informed consent, and the study has been approved by Ethics Committee of the University of Leipzig.

**Lifestyle – Immune System – Allergy Study and German Infant Study on the influence of Nutrition Intervention (LISA+GINI)**

*Study Population*

The influence of Life-style factors on the development of the Immune System and Allergies in East and West (LISA) Study is a population based birth cohort study. A total of 3094 healthy, full-term neonates were recruited between 1997 and 1999 in Munich, Leipzig, Wesel and Bad Honnef (35). The participants were not pre-selected based on family history of allergic diseases.

A total of 5991 mothers and their newborns were recruited into the German Infant study on the influence of Nutrition Intervention PLUS environmental and genetic influences on allergy development (GINIplus) between September 1995 and June 1998 in Munich and Wesel (36). Infants with at least one allergic parent and/or sibling were allocated to the interventional study arm investigating the effect of different hydrolysed formulas for allergy prevention in the first year of life. All children without a family history of allergic diseases and children whose parents did not give consent for the intervention were allocated to the non-interventional arm. Detailed descriptions of the LISA and GINIplus studies have been published elsewhere (35, 36). DNA was collected at the age 6 and 10 years. For both studies, approval by the local Ethics Committees and written consent from participant’s families were obtained.

*Phenotype*

Information on head circumference, height, and gestational age was collected from health records kept by the parents of the study participants using self-administered questionnaires completed by the parents.

*Genotyping and imputation*

1511 children from Munich from both studies were included (835 (55%) children from the GINIplus study and 676 (45%) children from the LISA study). 1423 individuals (835 from the GINIplus study and 588 from the LISA study) were analyzed using the Affymetrix Human SNP Array 5.0 and 88 individuals from the LISA study were analyzed using Affymetrix Human SNP Array 6.0. Genotypes were called using BRLMM-P algorithm (5.0), respectively BIRDSEED V2 algorithm (6.0). In each of the two data sets, criteria for exclusion of individuals were: a call rate below 95%, a heterozygosity outside mean +/- 4sd, a failure of the sex check or a failure of the similarity quality control using MDS analysis based on IBS. Criteria for exclusion of variants were: a call rate below 95%, a MAF < 0.01 and a HWE p-value < 0.00001. The filtered data sets were prephased using SHAPEIT V2 and imputation was done using IMPUTE2.3 considering the haplotypes from the 1000 Genomes Project Phase I v3 as a reference (March 2012 release, updated version from 26 Aug 2012, all ancestries, limited to variants with more than one minor allele copy).  **Norwegian Mother and Child (MoBa) Cohort Study  (Pål R. Njølstad)
*Study population***

The Norwegian Mother and Child Cohort Study is an open-ended cohort study that recruited pregnant women in Norway from 1999 to 2008. Approximately 114,000 children, 95,000 mothers, and 75,000 fathers of predominantly Norwegian ancestry were enrolled in the study from 50 hospitals all across Norway (37). Anthropometric measurements of the children were carried out at hospitals (at birth) and during routine visits by trained nurses at 6 weeks, 3, 6, 8 months, and 1, 1.5, 2, 3, 5, 7, and 8 years of age. Parents later transcribed these measurements to questionnaires. In 2012, the project Better Health By Harvesting Biobanks (HARVEST) randomly selected 11,490 umbilical cord blood DNA samples from the Norwegian Mother and Child Cohort Study’s biobank for genotyping, excluding samples matching any of the following criteria: (1) stillborn, (2) deceased, (3) twins, (4) non-existing Medical Birth Registry data, (5) missing anthropometric measurements at birth in Medical Birth Registry, (6) pregnancies where the mother did not answer the first questionnaire (as a proxy for higher fallout rate), and (7) missing parental DNA samples.

***Genotyping***

Genotyping was performed using Illumina’s HumanCoreExome-12 v.1.1 and HumanCoreExome-24 v.1.0 arrays for 6,938 and 4,552 samples, respectively, at the Genomics Core Facility located at the Norwegian University of Science and Technology, Trondheim, Norway. We used the Genome Reference Consortium Human Build 37 (GRCh37) reference genome for all annotations and included autosomal markers only for this study.

Genotypes were called in Illumina Genome Studio (v.2011.1). Cluster positions were identified from samples with call rate ≥ 0.98 and GenCall score ≥ 0.15. We excluded variants with low call rates, signal intensity, quality scores, heterozygote excess, and deviation from Hardy-Weinberg equilibrium (HWE) based on the following QC parameters: call rate < 98%, cluster separation < 0.4, 10% GC-score < 0.3, AA T Dev > 0.025, HWE *P*-value < 10^-6^. Samples were excluded based on call rate < 98% and heterozygosity excess > 4 SD. Study participants with non-Norwegian ancestry were excluded after merging with samples from the HapMap project (ver. 3). Sample pairs with PI_HAT > 0.1 in identical-by-descent (IBD) calculations were resolved by removing a random sample in each pair. After genotype calling and QC, 9,286 samples (80.8%) remained eligible for analysis.

***Pre-phasing and imputation***

Prior to imputation, insertions and deletions were removed to make the dataset congruent with Haplotype Reference Consortium (HRC) v.1.1 imputation panel using the HRC Imputation preparation tool by Will Rayner version 4.2.5 (www.well.ox.ac.uk/~wrayner/tools). Allele, marker position, and strand orientation were updated to match the reference panel. A total of 384,855 markers remained eligible for phasing and imputation. Pre-phasing was conducted locally using Shapeit v2.790 (38). Imputation was performed at the Sanger Imputation Server (see URLs) with positional Burrows-Wheeler transform (39) and HRC version 1.1 as reference panel.

***Phenotypes***

Age and head circumference values were extracted from hospital records through the Norwegian Medical Birth Registry for measurements at birth and from the study questionnaires for remaining time points. Pregnancy duration in days was extracted from Medical Birth Registry and pregnancies with duration < 37 weeks 0 day were excluded (515 pregnancies). Extreme outliers, typically an error in handwritten text parsing or a consequence of incorrect units, were excluded, and values at birth and one year of age were extracted for standardization and genome wide analyses. The quality control of the phenotypes was conducted in R version 3.5.1 (2018-07-02) -- "Feather Spray" (www.R-project.org).

***Statistical analyses***

Genome-wide analyses were performed using SNPTEST v.2.5.2 (mathgen.stats.ox.ac.uk/genetics_software/snptest/snptest.html) using dosages of alternate allele with an additive linear model using sex, batch, and ten principal components as covariates.

**Norwegian Mother and Child (MoBa) Cohort Study  (Bo Jacobsson)**

***Study population***

From the MoBa target population described above, 3,121 samples of mothers and children were genotyped. In addition to the previous criteria, samples were selected from two strata of gestational age: 154-258 d (preterm) and 273-286 d (full term). The preterm group was used for QC and imputation, but otherwise not analyzed in this study.

***Genotyping***

Genotyping was performed using Illumina Human660W-Quad BeadChip array. Genotypes were called in Illumina Genome Studio with standard exclusion criteria. We excluded variants with call rates < 97%, HWE *P*-value < 10^-7^ based on full-term samples (mothers-only for X chromosome). Samples were excluded based on call rate < 97% and heterozygosity excess > 3 SD. Study participants with non-Norwegian ancestry were excluded by merging with HapMap samples and inspecting the first 3 PCs; Euclidean distance of >10SD was taken as the threshold.

***Phasing and imputation***

Pre-phasing and imputation were conducted locally using Shapeit v2.r790 (38), with --duohmm flag for related-sample phasing. Haplotype reference was obtained from the 1000 Genomes Project, March 2012 version. Imputation was performed with same reference panel, using Impute2 v2.31. Pseudo-autosomal regions of the X chromosome were not imputed.

***Phenotypes***

Age and head circumference values were extracted as for the MoBa cohort above. Pregnancies with duration < 37 weeks 0 day were excluded. Extreme outliers were excluded based on visual inspection of (gestational) age vs phenotype, and values at birth and one year of age were extracted for standardization and genome wide analyses.

***Statistical analyses***

Genome-wide analyses were performed using SNPTEST v.2.5.2 (mathgen.stats.ox.ac.uk/genetics_software/snptest/snptest.html) using dosages of alternate allele with an additive linear model.

**Northern Finnish Birth Cohort Study 1966 (NFBC66)**The Northern Finland Birth Cohort study 1966 (NFBC1966) (http://www.oulu.fi/nfbc/) includes 12,058 live born individuals, of European descent, with expected dates of birth during 1966 in the two northernmost provinces of Finland, Oulu and Lapland (40). The University of Oulu Ethics Committee and the Ethical Committee of Northern Ostrobothnia Hospital District have approved the study. The data on all cohort members were prospectively collected since pregnancy and supplemented at the ages of 1, 14, 31 and 46 years. Growth measurements were obtained from communal child health clinics (41). All those living in northern Finland or in the capital area were invited to a clinical examination and blood sampling at age 31 years (42).
 DNA was extracted from 5402 individuals and GWAS data for current analyses. Illumina’s HumanCNV370-Duo DNA Analysis BeadChip was used to obtain genome-wide data. It contains an informative set of tag SNPs derived from the HapMap European-derived (CEU) sample. Imputation was performed on 328,007 SNPs using IMPUTE software version 0.3.1, applying information threshold of >0.4 and MAF threshold of >1%. **Northern Finland Birth Cohort Study 1986 (NFBC86)**

The NFBC1986 includes 9,432 live born children with expected dates of birth between 1st July 1985 and 30th June 1986 in the two northernmost provinces of Finland, Oulu and Lapland. The University of Oulu Ethics Committee and the Ethical Committee of Northern Ostrobothnia Hospital District have approved the study. The cohort has been followed up since early pregnancy until young adulthood. Growth measurements were obtained from communal child health clinics. All those alive with known address were invited to a clinical examination at the age of 15 to 16 years. At this age, blood samples were drawn and DNA was extracted for 6,266 subjects using standard methods. Participants provided written informed consent.

**The Physical Activity and Nutrition in Children (PANIC) Study**

The Physical Activity and Nutrition in Children (PANIC) Study is an 8-year controlled physical activity and dietary intervention study in a population sample of children from the city of Kuopio, Finland (<http://www.panicstudy.fi/en/panic-study-briefly>) (43). The main aims of the study are to investigate behavioral, biological, environmental, and genetic risk factors for overweight, type 2 diabetes, atherosclerotic cardiovascular diseases, musculoskeletal diseases, psychic problems, dementia, and oral health problems and the effects of a long-term physical activity and dietary intervention on risk factors for these chronic diseases and conditions. Altogether 512 children 6-9 years of age participated in the baseline examinations in 2007-2009. Six children were excluded from the study at baseline because of physical disabilities that could hamper participation in the intervention or no time or motivation to attend in the study. The remaining 506 children were divided in the physical activity and dietary intervention group and the control group. The intervention included six physical activity and dietary counseling sessions for the children and their parents or caregivers during the 2-year follow-up (0.5, 1.5, 3, 6, 12, and 18 months after baseline) (http://www.panicstudy.fi/en/intervention). The control group received general advice on health improving physical activity and diet according to the Finnish recommendations at baseline but no active intervention. Altogether 440 (87%) of the 506 children participated in the 2-year follow-up examinations in 2009-2011. The intervention was continued between 2-year and 8-year follow-up examinations and included seven physical activity and dietary counseling sessions (24, 36, 48, 60, 72, 84, and 96 months after baseline). Altogether 278 adolescents participated in the 8-year follow-up examinations in 2016-2017. A large number of behavioral, biological, environmental, and genetic risk factors for obesity, type 2 diabetes, atherosclerotic cardiovascular diseases, musculoskeletal diseases, psychic problems, dementia, and oral health problems were assessed between fetal period and adolescence (http://www.panicstudy.fi/en/assessments1). Most of the assessments were performed at baseline in 2007-2009, at 2-year follow-up in 2009-2011, and at 8-year follow-up in 2016-2017. The most important assessments will also be repeated at 13-year follow-up in 2021-2023. The PANIC study protocol was approved by the Research Ethics Committee of the Hospital District of Northern Savo. A written informed consent was acquired from the parents or caregivers of the children, and the children also provided their assent to participation.

The present analyses were carried out among children of European ethnic origin with genome-wide data. Birth and early-life head circumference were obtained from maternity health records from 452 and 370 children, respectively. Genomic DNA was isolated from the blood mononuclear cells using the QIAamp DNA Blood kit (Qiagen, Hilden, Germany). Genotyping was performed using the Illumina Custom Infinium CardioMetabo BeadChip (Illumina, San Diego, CA, USA) in the discovery phase and the Illumina Infinium HumanCoreExome BeadChip in the replication phase (Illumina, San Diego, CA, USA).

**Prevention and incidence of asthma and mite allergy birth cohort study - (PIAMA)**PIAMA is a birth cohort study consisting of two parts: a placebo controlled intervention study in which the effect of mite impermeable mattress covers on the development of asthma and allergy was studied and a natural history study in which no intervention took place. Details of the study design have been published previously (44). Recruitment took place in 1996-1997 through prenatal clinics. A screening questionnaire was distributed to pregnant women visiting one of 52 prenatal clinics at three regions in the Netherlands. A total of 10,232 pregnant women completed a validated screening questionnaire. Mothers reporting a history of asthma, current hay fever or allergy to pets or house dust mite were defined as allergic. Based on this screening, 7,862 women were invited to participate, of whom 4,146 women (1,327 allergic and 2,819 non-allergic) gave written informed consent. Follow-up of the children took place at 3 months of age and annually from 1 to 8 years of age. The Medical Ethical Committees of the participating institutes approved the study, and all participants gave written informed consent. Head circumference at birth was collected with a questionnaire when the children were 3 months of age. Parents were asked to enter the head circumference at birth as reported in the delivery report. DNA was collected from 2,162 children. Genome-wide genotyping was performed in two phases. The first phase was performed within the framework of the GABRIEL Consortium using an Illumina Human 610K quad array (45). Genotypes were available from 172 children with asthma and from 187 controls after quality control. A second group of 268 children who were more extensively examined during follow up was genotyped with an Illumina HumanOmniExpress array. A final group of 1377 children was genotyped with the Illumina Human Omni Express Exome Array.  The current analysis was restricted to individuals of European ethnic origin with genome-wide data and phenotype information (n=917 children).

**The Raine Study**The Raine Study is a prospective pregnancy cohort where 2900 mothers where recruited between 1989 and 1991. Recruitment took place at Western Australia’s major perinatal centre, King Edward Memorial Hospital, and nearby private practices (46, 47). Women who had sufficient English language skills, an expectation to deliver at King Edward Memorial Hospital, and an intention to reside in Western Australia to allow for future follow-up of their child were eligible for the study.

The Raine Study is known to be one of the largest successfully prospective cohorts richly phenotyped at multiple time points over pregnancy, infancy, childhood adolescence, and young adult. The mothers completed questionnaires regarding their children and the children had physical examinations at ages 1, 2, 3, 6, 8, 10, 14, 17, 20 and 22 years. In the present work, information on head circumference was obtained at birth and at the first year follow-up and recorded to the nearest 0.1cm. Prior to participation, written informed consent was obtained from all parents of the Raine Study participants (Gen2).

The Gen2 of the Raine Study participants were genotyped using the Illumina Human660W-Quad BeadChip. Genotypes were called using the GenomeStudio software. We excluded closely related individuals and samples with extreme inbreeding coefficients, mislabelled gender or call rate < 97% and duplicates, leaving 1494 participants individuals who passed all quality control criteria. We applied a >95% genotype call rate filter for the inclusion of SNPs. Additional genotypes were imputed into 1000 Genomes Phase 1 using Mach and Mini--mac.

**SKOT (1 & 2)**
The SKOT study is an observational cohort study, monitoring infants from the age of 9 months to 36 months. Recruitment and inclusion criteria have been described in detail previously (48-50). In short, the 329 children included in SKOT 1 were healthy singletons randomly recruited from the National Civil Registry and living in Copenhagen or Frederiksberg municipality, Denmark, in 2006-2007 (48). The included children were born at term and had Danish-speaking parents. The 184 children included in SKOT 2 met all above criteria with the exception that they were recruited in 2010-2012 and were offspring of overweight mothers (with a pre-pregnancy BMI above 30kg/m^2^), who had participated in the *Treatment of Obese Pregnant Women* intervention study at Hvidore Hospital, Hvidovre (Denmark) (51). Prior to participation, written informed consent was obtained from all parents of the children included in SKOT 1 and SKOT 2. The Committees on Biomedical Research Ethics for the Capital Region of Denmark approved the study protocol of SKOT 1 (H-KF-2007-0003) and SKOT 2 (H-3-2010-122). Both studies were conducted in accordance with the principles of the Declaration of Helsinki.

Information on head circumference was obtained at birth and during the 9 months examination using a non-flexible tape and recorded to the nearest millimeter. Measurements were performed in triplicate and the average of the measurements was used in the statistical analysis. Children in SKOT 1 and 2 were genotyped using the Illumina Infinium HumanCoreExome Beadchip (Illumina, San Diego, CA, USA). Genotypes were called using the Genotyping module, Version 1.9.4 of GenomeStudio software, Version 2011.1 (Illumina). We applied a >95% genotype call rate filter for the inclusion of SNPs. We excluded closely related individuals and samples with extreme inbreeding coefficients, mislabelled gender or call rate < 95%, duplicates and individuals identified as ethnic outliers, leaving 275 individuals of SKOT 1 and 116 individuals of SKOT 2 individuals who passed all quality control criteria. Additional genotypes were imputed into 1000 Genomes Phase 1 using Impute 2. 

**Special Turku Coronary Risk Factor Intervention Project (STRIP)**

The STRIP study is a prospective randomised life-style intervention trial that began in infancy and continued through childhood and adolescence to early adulthood (52) Altogether 1,062 children born in 1989-1991 were recruited at the age of 5 months by the well-baby clinics in Turku, and were randomised into an intervention group (n=540) or a control group (n=522). The life-style intervention continued until the participants reached the age of 20 years (n=~500).

The purpose of the life-style intervention was to reduce exposure to cardiovascular disease risk factors with main focus on diet. Primary target of the dietary counselling was replacement of saturated fat with unsaturated fat in the child’s diet. The counselling also promoted intake of vegetables, fruits, and whole-grain products, and low intake of salt. In addition to investigating the efficiency of dietary counselling in improving risk factor levels, safety of the low-saturated fat diet was assessed in terms of growth and development. The main outcome measures comprise nutrient intake, serum lipid and lipoprotein concentrations, blood pressure, measures of somatic growth and development, and ultrasonic measures of arterial intima-media thickness, elasticity and endothelial function.

Head circumference at birth was obtained from maternity health records. Early-life head circumference was measured during study visit by a physician.

Altogether, 666 STRIP children were genotyped using the custom Illumina genotyping array, Metabochip, at the Center for Inherited Disease Research, the Johns Hopkins University, USA. Genotype imputation was performed using IMPUTE2 (23, 53) and a reference panel from the 1000 Genomes.
 The study was approved by the Joint Commission on Ethics of the Turku University and the Turku University Central Hospital. Informed consent was obtained from all parents at the beginning of the trial and from the children at 15 and 18 years of age.

**TDCOB**

The Danish Childhood Obesity Biobank (ClinicalTrials.gov identifier NCT00928473) comprises data from children and adolescents with normal weight, overweight, or obesity. Data material was collected between January 2009 and March 2015. A total of 1,069 children and adolescents (aged 6‐18 years) with overweight or obesity were recruited through The Children’s Obesity Clinic, Department of Pediatrics, Copenhagen University Hospital Holbæk in Denmark. Overweight was defined as a BMI above the 90th percentile (BMI SDS > 1.28) according to age and sex in a Danish reference population (54). Between September 2010 and March 2013, a population‐based control sample of 719 Danish children and adolescents aged 6 to 18 years was recruited from schools across 11 municipalities in Denmark. This study was conducted in accordance with the Helsinki Declaration of 1983. An informed written and oral consent was obtained from all participants or from their parents if the participant was younger than 18 years of age. This study was approved by the Ethics Committee of Region Zealand, Denmark (ID number SJ‐104), and by the Danish Data Protection Agency.

  Genotyping was performed on 1,788 individuals using the Illumina Infinium HumanCoreExome BeadChip (Illumina, San Diego, California). Genotypes were called using the Genotyping module (version 1.9.4) of GenomeStudio Software (version 2011.1; Illumina). We excluded individuals identified as duplicates, ethnic outliers, or with extreme inbreeding coefficients, mislabeled gender, or a call‐rate < 95%, leaving 1,618 individuals (*n*overweight/obesity = 920, *n*population‐based sample = 698) who passed all quality control criteria. Additional genotypes were imputed into the 1000 Genomes phase 1 panel using IMPUTE2.

**TEENS of Attica: Genes and Environment Study (TEENAGE)**

The TEENAGE study is a cross-sectional study. The study target population comprised 857 adolescent students aged 13–15 years attending the first three classes of public secondary schools located in the wider Athens area of Attica. Prior to recruitment all study participants gave their verbal assent along with their parents’/guardians’ written consent forms. The study protocol was approved by the Institutional Review Board of Harokopio University and the Greek Ministry of Education, Lifelong Learning and Religious Affairs (55). Participants’ birth and early-life head circumference were derived from their medical records. All deliveries before 37 completed weeks of gestation were excluded from all analyses. TEENAGE DNA samples were genotyped using Illumina HumanOmniExpress BeadChips (Illumina, San Diego, USA) at the Welcome Trust Sanger Institute, Hinxton, UK (56) DNA samples of 707 study participants were genotyped using Illumina HumanOmniExpress BeadChips (Illumina, San Diego, CA, USA) at the Wellcome Trust Sanger Institute, Hinxton, UK. Genotyping and data quality control have been described previously (56). Genotypes were called using Illuminus algorithm (57) and SNPs were imputed using the program IMPUTE (24).

**UK Biobank**

*Study population*

The UK Biobank (<http://www.ukbiobank.ac.uk>) is a prospective cohort study that recruited approximately 500,000 people aged 40-69 from across the United Kingdom between 2006 and 2010. Participants have undergone measures, provided blood, urine and saliva samples for future analysis, detailed information about themselves and agreed to have their health followed. Ethical approval for UK Biobank was received from the research ethics committee (REC reference 11/NW/0382). Informed consent was provided by all participants.

*Genotyping*The UK Biobank genotyping procedure has been described elsewhere (58). In short, two custom genotyping arrays (UK BiLEVE Axiom Array and UK Biobank Axiom Array) were used to genotype 488,377 individuals (58), and were subsequently imputed to the Haplotype Reference Consortium (HRC) version 1.1 reference panel. We only included unrelated participants with self-reported ‘White British’ ancestry, without more than ten putative third-degree relatives in the kinship table, samples that were not identified as outliers in heterozygosity and missing rates, that were not identified as putatively carrying sex chromosome configurations.

*Phenotyping*A subset of UK Biobank participants underwent magnetic resonance imaging (MRI) of the brain on a standard Siemens Skyra 3.0 T scanner (Siemens Medical Solutions, Germany). From the T1 sequences, intracranial volume was estimated using the FreeSurfer 6.0 software.

**Methods** Study design birth head circumference

**Study design**We conducted a two-stage meta-analysis in children of European ancestry to identify genetic loci associated with birth and early-life head circumference. Sex- and gestational age-adjusted standard deviation scores (SDS) were created for birth head circumference using Growth Analyzer 3.0 across all studies (59). In the case of twin pairs and siblings, only one of each twin or sibling pair was included, either randomly or based on genotyping or imputation quality.

In the discovery stage, we performed a meta-analysis of 22 studies (N= 32,084), including the Avon Longitudinal Study of Parents and Children (ALSPAC, N= 5,690), Children’s Hospital of Philadelphia (CHOP, N= 300), the Copenhagen Prospective Studies on Asthma in Childhood 2000 (COPSAC2000, N= 345), 2010 (COPSAC2010, N= 594), and Registry (COPSAC Registry, N= 441), the Danish National Birth Cohort- preterm birth study (DNBC-PTB, N= 989), the Generation R Study (GenerationR, N= 1,859), Lifestyle – Immune System – Allergy Study and German Infant Study on the influence of Nutrition Intervention (LISA+GINI, N= 1,350), the Genetics of Overweight Young Adults Study (DNBC GOYA-offspring, N= 895), HAPO (N= 1,322), the INfancia y Medio Ambiente [Environment and Childhood] Project, with two subcohorts that were entered into the meta-analysis together (INMA-Menorca, Sabadell and Valencia subcohort (N= 942), the Leipzig Research Center for Civilization Diseases - Child study (LIFE-Child, N= 624), the Norwegian Mother Child Cohort (MoBa, N= 579), the Norwegian Mother Child Cohort (MoBa, N= 9,193), the Northern Finland Birth Cohort 1986 (NFBC 1986, N= 1,398), the Physical Activity and Nutrition in Children Study (PANIC, N= 452), the Raine Study (Raine Study, N= 1,344), the Småbørns Kost Og Trivsel study, including two subcohorts (SKOT 1, N= 167 and SKOT 2, N= 112), the Special Turku Coronary Risk factor Intervention Project (STRIP, N= 584), The Danish Childhood Obesity Biobank (TDCOB, N= 1,208), and the TEENs of Attica: Genes and Environment (TEENAGE, N= 197).

In the replication stage, we included 6 studies (N= 3,750): The European Childhood Obesity Project (CHOP Study, N= 361), The Exeter Family Study of Childhood Health (EFSOCH, N= 622), The Helsinki Birth Cohort Study (HBCS, N= 1,551), 383 additional children from the the INfancia y Medio Ambiente [Environment and Childhood] Project (INMA- Gipuzkoa subcohort, N= 383), the Prevention and incidence of asthma and mite allergy birth cohort study (PIAMA, N= 917), Project Viva (N= 324), and Isle of Wight 3rdGeneration Study (IOW, N= 92).Characteristics of discovery and replication studies can be found in **Table S1**. The study design of early-life head circumference can be found in the main manuscript.

**References**

1. Ikram MA, Fornage M, Smith AV, Seshadri S, Schmidt R, Debette S, et al. Common variants at 6q22 and 17q21 are associated with intracranial volume. Nat Genet. 2012;44(5):539-44.

2. Savage JE, Jansen PR, Stringer S, Watanabe K, Bryois J, de Leeuw CA, et al. Genome-wide association meta-analysis in 269,867 individuals identifies new genetic and functional links to intelligence. Nat Genet. 2018;50(7):912-9.

3. Jansen IE, Savage JE, Watanabe K, Bryois J, Williams DM, Steinberg S, et al. Genome-wide meta-analysis identifies new loci and functional pathways influencing Alzheimer's disease risk. Nat Genet. 2019;51(3):404-13.

4. Nagel M, Jansen PR, Stringer S, Watanabe K, de Leeuw CA, Bryois J, et al. Meta-analysis of genome-wide association studies for neuroticism in 449,484 individuals identifies novel genetic loci and pathways. Nat Genet. 2018;50(7):920-7.

5. Lee JJ, Wedow R, Okbay A, Kong E, Maghzian O, Zacher M, et al. Gene discovery and polygenic prediction from a genome-wide association study of educational attainment in 1.1 million individuals. Nat Genet. 2018;50(8):1112-21.

6. van Eijsden M, Vrijkotte TG, Gemke RJ, van der Wal MF. Cohort profile: the Amsterdam Born Children and their Development (ABCD) study. Int J Epidemiol. 2011;40(5):1176-86.

7. Lane JA, Noble JA. Maximizing deoxyribonucleic acid yield from dried blood spots. J Diabetes Sci Technol. 2010;4(2):250-4.

8. Boyd A, Golding J, Macleod J, Lawlor DA, Fraser A, Henderson J, et al. Cohort Profile: the 'children of the 90s'--the index offspring of the Avon Longitudinal Study of Parents and Children. Int J Epidemiol. 2013;42(1):111-27.

9. Fraser A, Macdonald-Wallis C, Tilling K, Boyd A, Golding J, Davey Smith G, et al. Cohort Profile: the Avon Longitudinal Study of Parents and Children: ALSPAC mothers cohort. Int J Epidemiol. 2013;42(1):97-110.

10. St Pourcain B, Robinson EB, Anttila V, Sullivan BB, Maller J, Golding J, et al. ASD and schizophrenia show distinct developmental profiles in common genetic overlap with population-based social communication difficulties. Mol Psychiatry. 2018;23(2):263-70.

11. Koletzko B, von Kries R, Closa R, Escribano J, Scaglioni S, Giovannini M, et al. Lower protein in infant formula is associated with lower weight up to age 2 y: a randomized clinical trial. Am J Clin Nutr. 2009;89(6):1836-45.

12. Weber M, Grote V, Closa-Monasterolo R, Escribano J, Langhendries JP, Dain E, et al. Lower protein content in infant formula reduces BMI and obesity risk at school age: follow-up of a randomized trial. Am J Clin Nutr. 2014;99(5):1041-51.

13. Rzehak P, Saffery R, Reischl E, Covic M, Wahl S, Grote V, et al. Maternal Smoking during Pregnancy and DNA-Methylation in Children at Age 5.5 Years: Epigenome-Wide-Analysis in the European Childhood Obesity Project (CHOP)-Study. PLoS One. 2016;11(5):e0155554.

14. Rzehak P, Covic M, Saffery R, Reischl E, Wahl S, Grote V, et al. DNA-Methylation and Body Composition in Preschool Children: Epigenome-Wide-Analysis in the European Childhood Obesity Project (CHOP)-Study. Sci Rep. 2017;7(1):14349.

15. Kirchberg FF, Harder U, Weber M, Grote V, Demmelmair H, Peissner W, et al. Dietary protein intake affects amino acid and acylcarnitine metabolism in infants aged 6 months. J Clin Endocrinol Metab. 2015;100(1):149-58.

16. Bisgaard H. The Copenhagen Prospective Study on Asthma in Childhood (COPSAC): design, rationale, and baseline data from a longitudinal birth cohort study. Ann Allergy Asthma Immunol. 2004;93(4):381-9.

17. Bisgaard H, Vissing NH, Carson CG, Bischoff AL, Folsgaard NV, Kreiner-Moller E, et al. Deep phenotyping of the unselected COPSAC2010 birth cohort study. Clin Exp Allergy. 2013;43(12):1384-94.

18. Bonnelykke K, Sleiman P, Nielsen K, Kreiner-Moller E, Mercader JM, Belgrave D, et al. A genome-wide association study identifies CDHR3 as a susceptibility locus for early childhood asthma with severe exacerbations. Nat Genet. 2014;46(1):51-5.

19. Loisel DA, Du G, Ahluwalia TS, Tisler CJ, Evans MD, Myers RA, et al. Genetic associations with viral respiratory illnesses and asthma control in children. Clin Exp Allergy. 2016;46(1):112-24.

20. Olsen J, Melbye M, Olsen SF, Sorensen TI, Aaby P, Andersen AM, et al. The Danish National Birth Cohort--its background, structure and aim. Scand J Public Health. 2001;29(4):300-7.

21. Genomes Project C, Abecasis GR, Altshuler D, Auton A, Brooks LD, Durbin RM, et al. A map of human genome variation from population-scale sequencing. Nature. 2010;467(7319):1061-73.

22. Delaneau O, Marchini J, Zagury JF. A linear complexity phasing method for thousands of genomes. Nat Methods. 2011;9(2):179-81.

23. Howie BN, Donnelly P, Marchini J. A flexible and accurate genotype imputation method for the next generation of genome-wide association studies. PLoS Genet. 2009;5(6):e1000529.

24. Marchini J, Howie B. Genotype imputation for genome-wide association studies. Nat Rev Genet. 2010;11(7):499-511.

25. Knight B, Shields BM, Hattersley AT. The Exeter Family Study of Childhood Health (EFSOCH): study protocol and methodology. Paediatr Perinat Epidemiol. 2006;20(2):172-9.

26. Manichaikul A, Mychaleckyj JC, Rich SS, Daly K, Sale M, Chen WM. Robust relationship inference in genome-wide association studies. Bioinformatics. 2010;26(22):2867-73.

27. Abraham G, Inouye M. Fast principal component analysis of large-scale genome-wide data. PLoS One. 2014;9(4):e93766.

28. Kooijman MN, Kruithof CJ, van Duijn CM, Duijts L, Franco OH, van IMH, et al. The Generation R Study: design and cohort update 2017. Eur J Epidemiol. 2016;31(12):1243-64.

29. Li Y, Willer C, Sanna S, Abecasis G. Genotype imputation. Annu Rev Genomics Hum Genet. 2009;10:387-406.

30. Li Y, Willer CJ, Ding J, Scheet P, Abecasis GR. MaCH: using sequence and genotype data to estimate haplotypes and unobserved genotypes. Genet Epidemiol. 2010;34(8):816-34.

31. Guxens M, Ballester F, Espada M, Fernandez MF, Grimalt JO, Ibarluzea J, et al. Cohort Profile: the INMA--INfancia y Medio Ambiente--(Environment and Childhood) Project. Int J Epidemiol. 2012;41(4):930-40.

32. Ferrer M, Garcia-Esteban R, Iniguez C, Costa O, Fernandez-Somoano A, Rodriguez-Delhi C, et al. Head circumference and child ADHD symptoms and cognitive functioning: results from a large population-based cohort study. Eur Child Adolesc Psychiatry. 2019;28(3):377-88.

33. Quante M, Hesse M, Dohnert M, Fuchs M, Hirsch C, Sergeyev E, et al. The LIFE child study: a life course approach to disease and health. BMC Public Health. 2012;12:1021.

34. Poulain T, Baber R, Vogel M, Pietzner D, Kirsten T, Jurkutat A, et al. The LIFE Child study: a population-based perinatal and pediatric cohort in Germany. Eur J Epidemiol. 2017;32(2):145-58.

35. Heinrich J, Bolte G, Holscher B, Douwes J, Lehmann I, Fahlbusch B, et al. Allergens and endotoxin on mothers' mattresses and total immunoglobulin E in cord blood of neonates. Eur Respir J. 2002;20(3):617-23.

36. Berg A, Kramer U, Link E, Bollrath C, Heinrich J, Brockow I, et al. Impact of early feeding on childhood eczema: development after nutritional intervention compared with the natural course - the GINIplus study up to the age of 6 years. Clin Exp Allergy. 2010;40(4):627-36.

37. Magnus P, Birke C, Vejrup K, Haugan A, Alsaker E, Daltveit AK, et al. Cohort Profile Update: The Norwegian Mother and Child Cohort Study (MoBa). Int J Epidemiol. 2016;45(2):382-8.

38. Delaneau O, Zagury JF, Marchini J. Improved whole-chromosome phasing for disease and population genetic studies. Nat Methods. 2013;10(1):5-6.

39. Durbin R. Efficient haplotype matching and storage using the positional Burrows-Wheeler transform (PBWT). Bioinformatics. 2014;30(9):1266-72.

40. Rantakallio P. Groups at risk in low birth weight infants and perinatal mortality. Acta Paediatr Scand. 1969;193:Suppl 193:1+.

41. Sovio U, Kaakinen M, Tzoulaki I, Das S, Ruokonen A, Pouta A, et al. How do changes in body mass index in infancy and childhood associate with cardiometabolic profile in adulthood? Findings from the Northern Finland Birth Cohort 1966 Study. Int J Obes (Lond). 2014;38(1):53-9.

42. Jarvelin MR, Sovio U, King V, Lauren L, Xu B, McCarthy MI, et al. Early life factors and blood pressure at age 31 years in the 1966 northern Finland birth cohort. Hypertension. 2004;44(6):838-46.

43. Eloranta AM, Lindi V, Schwab U, Tompuri T, Kiiskinen S, Lakka HM, et al. Dietary factors associated with overweight and body adiposity in Finnish children aged 6-8 years: the PANIC Study. Int J Obes (Lond). 2012;36(7):950-5.

44. Brunekreef B, Smit J, de Jongste J, Neijens H, Gerritsen J, Postma D, et al. The prevention and incidence of asthma and mite allergy (PIAMA) birth cohort study: design and first results. Pediatr Allergy Immunol. 2002;13(s15):55-60.

45. Moffatt MF, Gut IG, Demenais F, Strachan DP, Bouzigon E, Heath S, et al. A large-scale, consortium-based genomewide association study of asthma. N Engl J Med. 2010;363(13):1211-21.

46. Straker L, Mountain J, Jacques A, White S, Smith A, Landau L, et al. Cohort Profile: The Western Australian Pregnancy Cohort (Raine) Study-Generation 2. Int J Epidemiol. 2017;46(5):1384-5j.

47. Newnham JP, Sharon SF, Michael CA, Stanley FJ, Landau LI. [Effects of frequent ultrasound during pregnancy: a randomised controlled trial] Effekter av frekvent ultraljud under graviditeten: en randomiserad kontrollerad studie. Jordemodern. 1994;107(3):83-6.
48. Madsen AL, Larnkjaer A, Molgaard C, Michaelsen KF. IGF-I and IGFBP-3 in healthy 9 month old infants from the SKOT cohort: breastfeeding, diet, and later obesity. Growth Horm IGF Res. 2011;21(4):199-204.
49. Andersen LB, Molgaard C, Michaelsen KF, Carlsen EM, Bro R, Pipper CB. Indicators of dietary patterns in Danish infants at 9 months of age. Food Nutr Res. 2015;59:27665.
50. Ejlerskov KT, Christensen LB, Ritz C, Jensen SM, Molgaard C, Michaelsen KF. The impact of early growth patterns and infant feeding on body composition at 3 years of age. Br J Nutr. 2015;114(2):316-27.
51. Renault KM, Norgaard K, Nilas L, Carlsen EM, Cortes D, Pryds O, et al. The Treatment of Obese Pregnant Women (TOP) study: a randomized controlled trial of the effect of physical activity intervention assessed by pedometer with or without dietary intervention in obese pregnant women. Am J Obstet Gynecol. 2014;210(2):134 e1-9.
52. Simell O, Niinikoski H, Ronnemaa T, Raitakari OT, Lagstrom H, Laurinen M, et al. Cohort Profile: the STRIP Study (Special Turku Coronary Risk Factor Intervention Project), an Infancy-onset Dietary and Life-style Intervention Trial. Int J Epidemiol. 2009;38(3):650-5.
53. Howie B, Fuchsberger C, Stephens M, Marchini J, Abecasis GR. Fast and accurate genotype imputation in genome-wide association studies through pre-phasing. Nat Genet. 2012;44(8):955-9.
54. Nysom K, Molgaard C, Hutchings B, Michaelsen KF. Body mass index of 0 to 45-y-old Danes: reference values and comparison with published European reference values. Int J Obes Relat Metab Disord. 2001;25(2):177-84.
55. Ntalla I, Giannakopoulou M, Vlachou P, Giannitsopoulou K, Gkesou V, Makridi C, et al. Body composition and eating behaviours in relation to dieting involvement in a sample of urban Greek adolescents from the TEENAGE (TEENs of Attica: Genes & Environment) study. Public Health Nutr. 2014;17(3):561-8.

56. Ntalla I, Panoutsopoulou K, Vlachou P, Southam L, William Rayner N, Zeggini E, et al. Replication of established common genetic variants for adult BMI and childhood obesity in Greek adolescents: the TEENAGE study. Ann Hum Genet. 2013;77(3):268-74.

57. Teo YY, Inouye M, Small KS, Gwilliam R, Deloukas P, Kwiatkowski DP, et al. A genotype calling algorithm for the Illumina BeadArray platform. Bioinformatics. 2007;23(20):2741-6.

58. Bycroft C, Freeman C, Petkova D, Band G, Elliott LT, Sharp K, et al. Genome-wide genetic data on ~500,000 UK Biobank participants. bioRxiv. 2017:166298.

59. Wikland KA, Luo ZC, Niklasson A, Karlberg J. Swedish population-based longitudinal reference values from birth to 18 years of age for height, weight and head circumference. Acta Paediatr. 2002;91(7):739-54.
